# Ischemic preconditioning affects phosphosites and accentuates myocardial stunning while reducing infarction size in rats

**DOI:** 10.3389/fcvm.2024.1376367

**Published:** 2024-03-15

**Authors:** Ahmed Elmahdy, Aaron Shekka Espinosa, Yalda Kakaei, Tetiana Pylova, Abhishek Jha, Ermir Zulfaj, Maryna Krasnikova, Amin Al-Awar, Zahra Sheybani, Valentyna Sevastianova, Evelin Berger, Amirali Nejat, Linnea Molander, Erik Axel Andersson, Elmir Omerovic, Shafaat Hussain, Björn Redfors

**Affiliations:** ^1^ Department of Molecular and Clinical Medicine, Institute of Medicine, Sahlgrenska Academy, University of Gothenburg, Gothenburg, Sweden; ^2^ Wallenberg Centre for Molecular and Translational Medicine, Institute of Medicine, University of Gothenburg, Gothenburg, Sweden; ^3^ Proteomics Core Facility, Sahlgrenska Academy, University of Gothenburg, Gothenburg, Sweden; ^4^ Department of Cardiology, Sahlgrenska University Hospital, Gothenburg, Sweden

**Keywords:** myocardial infarction, myocardial stunning, ischemic preconditioning, echocardiography, speckle tracking analysis, phosphoproteomics

## Abstract

**Background and aims:**

Ischemic preconditioning (IPC), i.e., brief periods of ischemia, protect the heart from subsequent prolonged ischemic injury, and reduces infarction size. Myocardial stunning refers to transient loss of contractility in the heart after myocardial ischemia that recovers without permanent damage. The relationship between IPC and myocardial stunning remains incompletely understood. This study aimed primarily to examine the effects of IPC on the relationship between ischemia duration, stunning, and infarct size in an ischemia-reperfusion injury model. Secondarily, this study aimed to examine to which extent the phosphoproteomic changes induced by IPC relate to myocardial contractile function.

**Methods and results:**

Rats were subjected to different durations of left anterior descending artery (LAD) occlusion, with or without preceding IPC. Echocardiograms were acquired to assess cardiac contraction in the affected myocardial segment. Infarction size was evaluated using triphenyl tetrazolium chloride staining. Phosphoproteomic analysis was performed in heart tissue from preconditioned and non-preconditioned animals. In contrast to rats without IPC, reversible akinesia was observed in a majority of the rats that were subjected to IPC and subsequently exposed to ischemia of 13.5 or 15 min of ischemia. Phosphoproteomic analysis revealed significant differential regulation of 786 phosphopeptides between IPC and non-IPC groups, with significant associations with the sarcomere, Z-disc, and actin binding.

**Conclusion:**

IPC induces changes in phosphosites of proteins involved in myocardial contraction; and both accentuates post-ischemic myocardial stunning and reduces infarct size.

## 1 Introduction

Brief episodes of myocardial ischemia prior to prolonged ischemia, so-called myocardial ischemic preconditioning (IPC), reduces infarct size in experimental models (1); but its precise mechanisms are incompletely understood and have not been reproduced in clinical medicine. In addition to inducing IPC, myocardial ischemia can induce myocardial stunning, i.e., the sudden and reversible loss of myocardial contractile function (2). Although myocardial IPC and stunning both occur after transient ischemia, they have been considered distinct phenomena (3).

Myocardial stunning has traditionally been thought of as a form of myocardial injury (4, 5); and it has been suggested that IPC may reduce or reverse myocardial stunning. However, numerous studies have failed to show any reduction in myocardial stunning by IPC (6).

Other studies have shown that myocardial stunning occurs within seconds of blood supply interruption, and precedes the depletion of the cardiomyocyte's energy stores (7). Since at least 80% of the myocardial energy is typically used by the contractile apparatus, an alternative hypothesis is that myocardial stunning preserves energy for vital cellular processes and protects cells from ischemic cell death (7, 8). If myocardial stunning confers cardioprotection, IPC could be expected to accentuate rather than reduce (or mitigate) myocardial stunning.

The primary aim of this study was to examine the impact of IPC on the relationship between ischemia duration, myocardial stunning and infarct size in an experimental model of ischemia-reperfusion injury. The secondary aim of the study was to examine to which extent the phosphoproteomic changes induced by IPC relate to contractile function.

## 2 Materials and methods

### 2.1 Rats

Male Sprague-Dawley rats (*n* = 68) aged between six to eight weeks and weighing 250–350 g were sourced from Janvier Labs (Le Genest-Saint-Isle, France) and given one week to acclimate at the Laboratory for Experimental Biomedicine (Gothenburg, Sweden) before surgeries. Throughout the acclimation and experimental periods, the rats were housed in a facility that maintained a temperature of 21 °C and a 12 h light/dark cycle. The rats had unrestricted access to standard laboratory fodder and water during this time.

### 2.2 Ischemia-reperfusion model

Rats were anesthetized with an intraperitoneal injection of Ketamine (120 mg/kg) and Xylazine (5 mg/kg) (9, 10). They were intubated endotracheally and ventilated with a small-animal ventilator (SAR-1000: CWE Inc, PA, USA). The levels of end-tidal CO_2_ were continuously monitored using a CapStar-100 End-Tidal CO_2_ monitor (CWE Inc) and kept between 5%–6%. A continuous infusion of ketamine and xylazine (0.125 mg/ml and 3 mg/ml, respectively) in Ringer solution was administered through cannulation of the lateral tail vein during the surgery to maintain surgical anesthesia. To induce myocardial ischemia, the left anterior descending artery (LAD) was temporarily ligated 3 to 4 mm caudally from its origin using a 6.0 suture (Ethicon Inc, NJ, USA), following access to the mediastinum via left thoracotomy at the 4th intercostal space. The LAD occlusions were confirmed by observing cardiac akinesia on echocardiography (ECHO), left ventricular blanching, and electrocardiogram (ECG) changes. At the end of the ischemic time, the LAD ligature was released, and reperfusion was confirmed visually.

### 2.3 Study design

A pilot study determined the ischemia duration at which substantial necrosis, defined as an infarct size of 10% of the area at risk, was observed on histology among rats who were not subjected to IPC as well as among rats that were subjected to IPC. The different durations of ischemia that were studied in subsequent experiments were based on the results of these pilot experiments.

**Figure 1:**
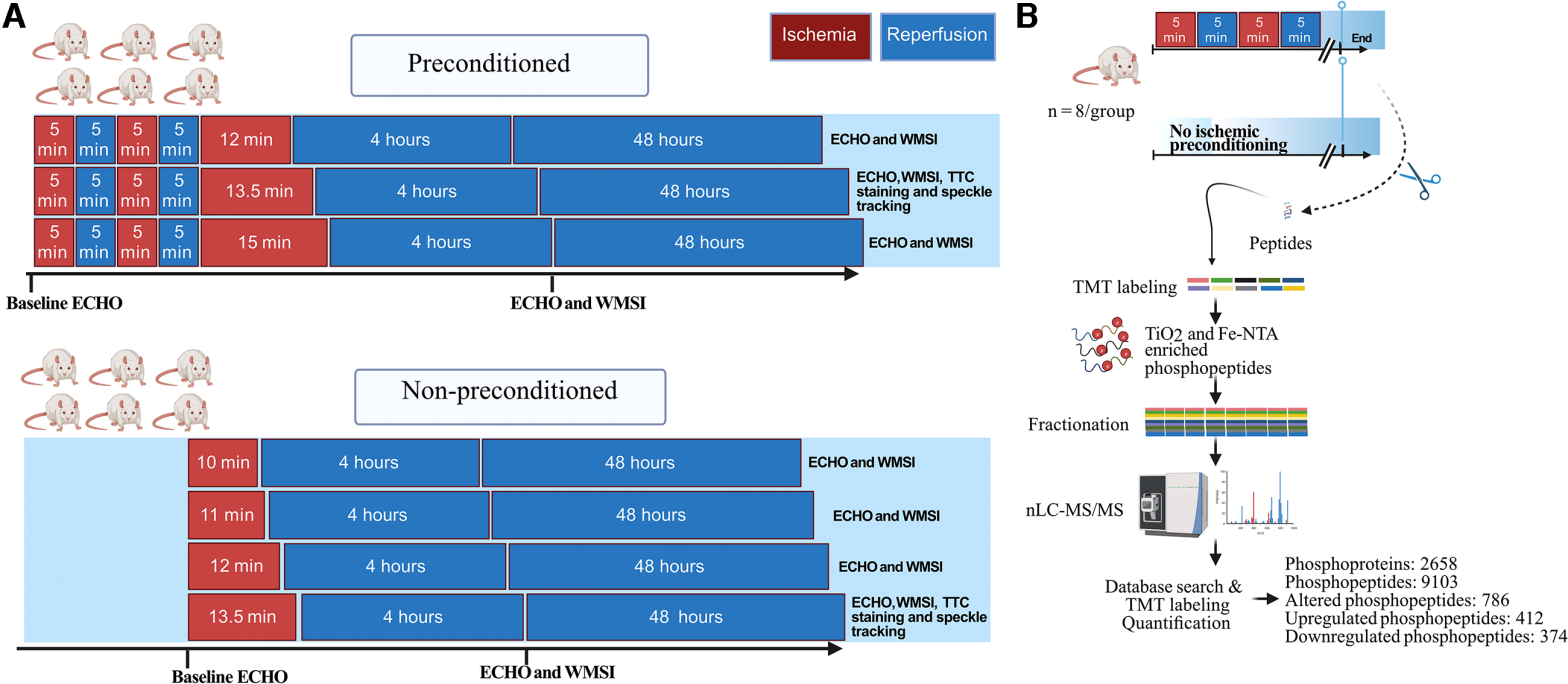
Study design. (**A**), Male rats (*n* = 6/group) were exposed to different durations of ischemia via left anterior descending coronary artery (LAD) occlusion, without or with cardiac ischemic preconditioning. Preconditioning was induced via 2 cycles of 5 min LAD occlusion interposed with 2 cycles of 5 min reperfusion. The wall motion score index (WMSI), measured on echocardiograms, was evaluated at baseline, 4 h, and 48 h. Based on these results, the 13.5 min time points were selected for further experiments and analysis with high-resolution speckle tracking and the infarct size was quantified with triphenyl tetrazolium chloride (TTC) staining. (**B**), Schematic representation of the phosphoproteomic analysis workflow using tissue from the anteroseptal wall from rats either subjected to ischemic preconditioning or not (*n* = 8/group). ECHO, echocardiography; TMT, tandem mass tags; nLC-MS/MS, nano-liquid chromatography tandem mass spectrometry. Figure created with Biorender.com.

To investigate the impact of IPC on the relationship between myocardial stunning and infarction after different ischemic times, the rats were allocated to IPC or no IPC (no ischemic preconditioning, NPC) followed by 10, 11, 12, 13,5 or 15 min of ischemia (index ischemia) (Figure 1A). The different durations of ischemia were selected to include ischemia durations that induced no necrosis (non-necrotic ischemia) either with or without IPC as well as durations that induced necrosis (necrotic ischemia) both with and without IPC. Rats that were allocated to IPC were subjected to two five-minute intervals of ischemia followed by 5 min of reperfusion prior to the ischemia.

In the first set of experiments, echocardiography was performed during the index ischemia and at 4 and 48 h after reperfusion. For each of the 7 groups (*n* = 6/group) we derived the proportion of rats that develop myocardial akinesia (defined as the lack of contraction in the affected segment) and the median (Q1, Q3) wall motion score index (WMSI) at both 4 and 48 h (11).

In the second set of experiments, rats were killed immediately following IPC (or NPC) and cardiac tissue from the affected myocardial was analyzed with phosphoproteomics (*n* = 8/group) (Figure 1B).

#### 2.3.1 Echocardiography

Echocardiography was performed using a VisualSonics 3,100 VEVO imaging station with a 15–30 MHz linear transducer (MX250; Fujifilm, Tokyo, Japan) and an integrated rail system to ensure consistent probe positioning. The imaging process involved obtaining an optimal parasternal long axis view, which allowed visualization of both the mitral and aortic valves as well as the maximum distance between the aortic valve and the cardiac apex. The cine loop of >1,000 frames was acquired using the ECG-gated kilohertz visualization technique. Additionally, a short axis view was obtained by rotating the probe 90 degrees and scanning to 6 mm below the mitral valve, perpendicularly on the long axis view.

#### 2.3.2 Wall motion score index

**Figure 2:**
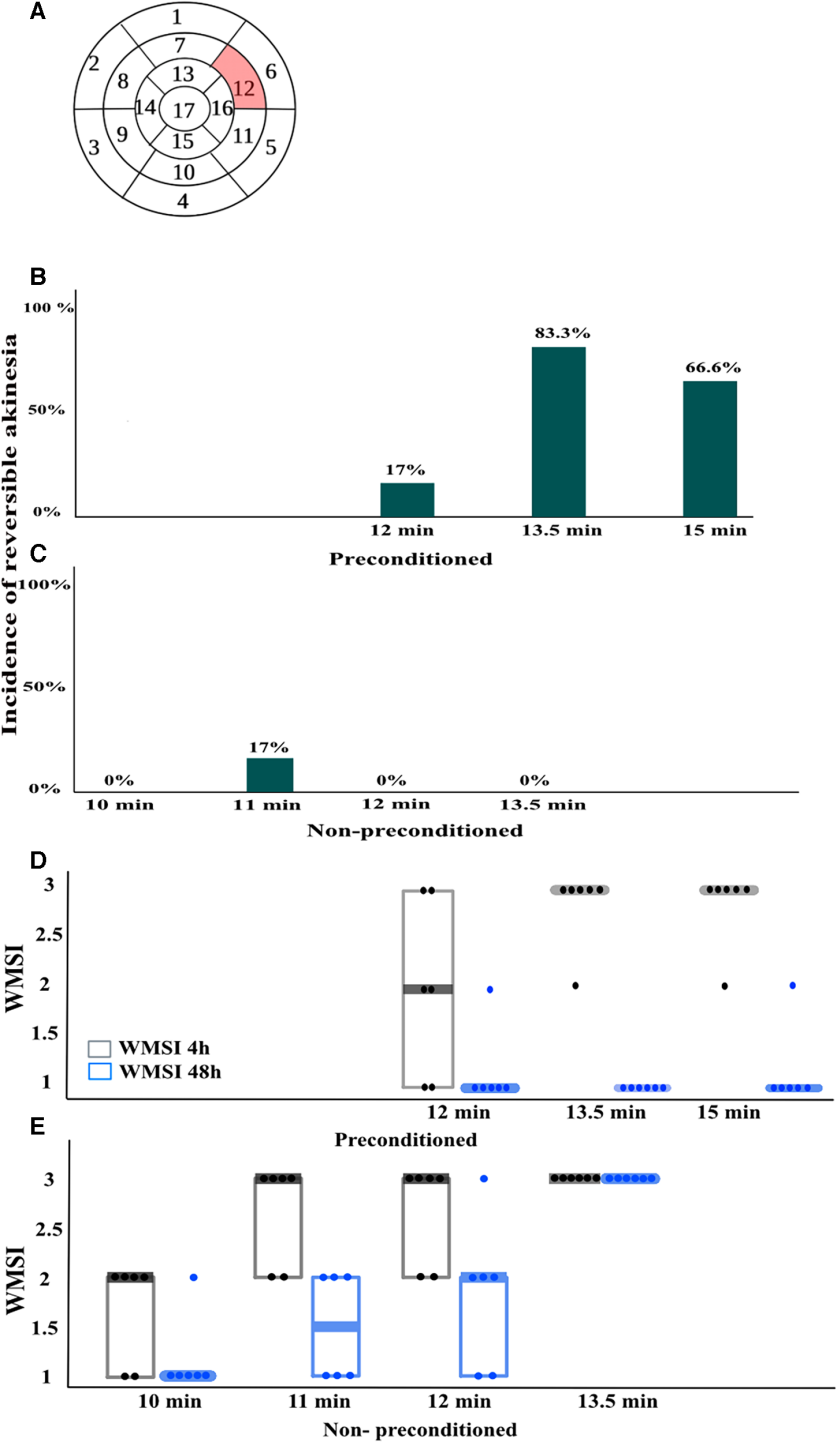
Wall motion score index (WMSI)(*n* = 6/group). (**A**), For WMSI, the left ventricle is divided into 17 segments and scored from 1 to 3 based on wall motion, where 1 is normal, 2 is hypokinesia, and 3 is akinesia. The highlighted segment 12 corresponds to the area exposed to ischemia during left anterior descending coronary artery (LAD) occlusion. Segment 12 was scored in echocardiograms taken at baseline, 4 h, and 48 h after LAD reperfusion. (**B,C**), the incidence of reversible akinesia, defined as the presence of akinesia at 4 h that was no longer present at 48 h in the different groups. (**D,E**), WMSI scores in different groups expressed as median (Q1, Q3). *n* = 6/group.

WMSI was used to assess regional wall motion abnormalities of the left ventricle in rats. The WMSI involves dividing the left ventricle into 17 segments and assigning a score to each segment based on the observed degree of wall motion abnormality. The scores are 1 (normal function), 2 (hypokinesia), and 3 (akinesia). The focus of the study was on segment 12 of the left ventricle, which corresponds to the anterolateral wall in the short-axis projection at 6 mm below the mitral valve (Figure 2A). This segment is supplied by the LAD and was subjected to ischemia during LAD ligation. Segment 12 was scored at baseline, 4 h, and 48 h, after reperfusion. To ensure the accuracy and reliability of the results, two experienced operators were blinded to evaluate the echocardiograms. Reversible myocardial akinesia was defined as the presence of akinesia at 4 h reperfusion with resolution of akinesia by 48 h.

#### 2.3.3 Speckle tracking analysis

**Figure 3:**
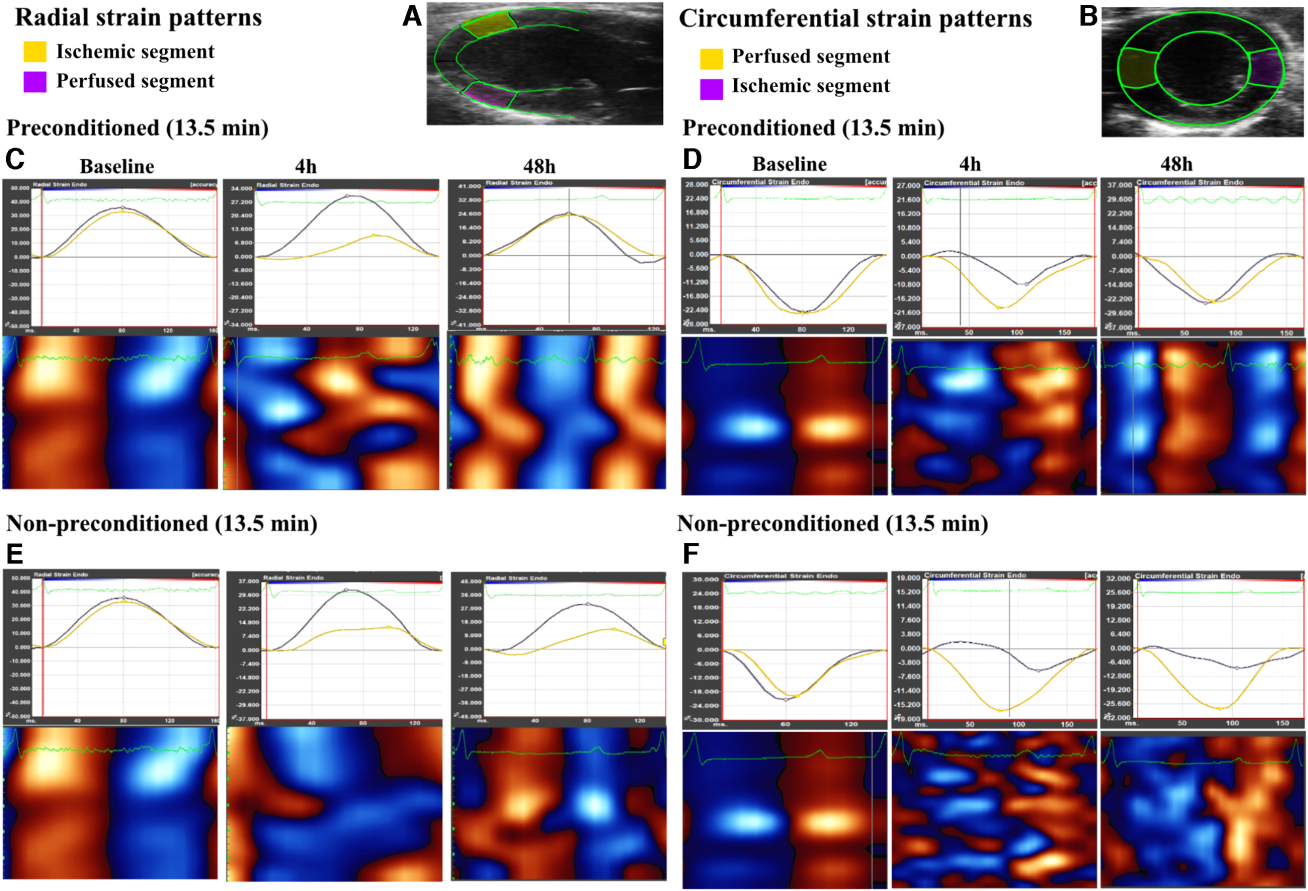
Radial and circumferential strain patterns observed in both ischemic and perfused segments over different time points, as well as heatmaps of the global wall motion. (**A,B**), representative echocardiogram for the long axis view and short axis view of the left ventricle, as traced during speckle tracking analysis with the ischemic and perfused control segments marked in yellow and pink respectively in the long axis while marked in pink and yellow respectively in the short axis. (**C,D**), strain patterns (radial and circumferential, respectively) illustrating a decline in peak strain and dyssynchrony in the ischemic segment as compared to the perfused segment at 4 h after left anterior descending artery (LAD) reperfusion, followed by recovery at 48 h in the preconditioned-13.5 min LAD occlusion group. Heatmaps show loss of systole (red in long axis and blue in short axis) and diastole (blue in long axis and red in short axis) regular cycle at 4 h, which had re-coordinated at 48 h. (**E,F**), strain patterns (radial and circumferential, respectively) in the non-preconditioned group-13.5 min LAD occlusion group, which exhibit a decline in peak strain and persistent dyssynchrony at both 4 h and 48 h in the ischemic segment. Heatmaps did not show re-coordinated systole-diastole cycle at 48 h.

Consecutive cardiac cycles were digitally acquired using the VevoStrain function in the Vevo Lab 5.7.1 software (Fujifilm). Considering the evaluations conducted by WMSI, both parasternal long-axis and mid-ventricular short-axis views were analyzed in IPC-13.5 and NPC-13.5 groups. The left ventricle was divided into 6 segments, and 48 sampling points were used to trace the endocardium to obtain strain measurements. The ischemic segments were identified as the anterior middle segment in the long-axis view and the anterior free wall segment in the short-axis view (Figures 3A,B). Longitudinal, radial, and circumferential peak strain (%) as well as time-to-peak strain (ms) were calculated in the ischemic segment, and followed serially at baseline, 4 h, and 48 h after LAD reperfusion. Additionally, general functional cardiac parameters such as left ventricular ejection fraction (LVEF), fractional shortening (FS), cardiac output (COP), fractional area change (FAC), and global longitudinal strain (GLS) were calculated and compared at baseline, 4 h, and 48 h after reperfusion in both groups.

### 2.4 Infarct size

Based on the WMSI-results, Triphenyl tetrazolium Chloride (TTC) staining was used to evaluate infarction size in a IPC-13.5 group (*n* = 5), representing myocardial stunning, and a non-preconditioned group (NPC-13.5, *n* = 5) in an acute experiment. The rationale behind conducting an acute experiment lies in the demonstrated optimal efficacy of TTC staining when employed shortly after the event, rather than an extended period thereafter (12). Myocardial infarction size was determined using Evans blue/TTC double staining as described in prior studies (9). Two hours after reperfusion, the LAD was re-ligated, and 5% Evans blue dye was injected via the lateral tail vein to label the non-ischemic region. After 1 min, the hearts were rapidly excised, cut by a scalpel into 5 transverse sections (2 mm thickness from apex to base) and incubated in 1% TTC for 10 min at 37°C in the dark. The tissue sections were then fixed in 10% formalin for 10 min, followed by a 10 min immersion in phosphate buffer (pH 7.4). The sections were weighed and mounted on glass slides, and images were acquired using a scanner (Seiko Epson, Nagano, Japan). Infarct size was calculated as a percentage of the overall infarct area to the overall area at risk i.e., the area not labeled by Evans blue dye, using ImageJ version 1.34. Two independent blinded investigators evaluated the infarcted and area-at-risk regions in each section.

### 2.5 Phosphoproteomic analysis

To explore the phosphoproteomic changes triggered by preconditioning ischemia, rats were randomly assigned into two groups: one group underwent ischemic preconditioning (*n* = 8), while the other group served as a control with no ischemic preconditioning (*n* = 8), as illustrated in Figure 1B, and cardiac tissue samples from the anteroseptal wall, specifically segment 12 (Figure 2A) were collected for phosphoproteomic analysis. Importantly, this time point corresponds to the moment just before inducing prolonged ischemia (as shown in Figure 1A).

#### 2.5.1 Sample preparation for phosphoproteomic analysis

Samples were homogenized on a FastPrep®-24 instrument (MP Biomedicals, OH, USA) for 5 repeated 40 s cycles at 6.5 m/s in lysis buffer containing 2% sodium dodecyl sulfate, 50 mM triethylammonium bicarbonate (TEAB). Lysed samples were centrifuged at 8,000 xg for 20 min and the supernatants were transferred to clean tubes. Protein concentrations were determined using Pierce™ BCA Protein Assay Kit (ThermoFisher Scientific) and a Benchmark™ Plus microplate reader (Bio-Rad Laboratories, Hercules, CA, USA) with bovine serum albumin solutions as standards.

Aliquots containing 200 µg of protein from each sample were incubated at room temperature for 60 min in the lysis buffer with DL-dithiothreitol at 100 mM final concentration. The reduced samples were processed using the modified filter-aided sample preparation method (13). Briefly, the samples were transferred to 30 kDa Microcon Centrifugal Filter Units (catalogue no. MRCF0R030, Merck), washed repeatedly with 8 M urea and once with digestion buffer [0.5% sodium deoxycholate (SDC) in 50 mM TEAB] prior to alkylation with 10 mM methyl methanethiosulfonate in digestion buffer for 30 min. Digestion was performed in digestion buffer by addition of Pierce MS grade Trypsin (ThermoFisher Scientific), in an enzyme to protein ratio of 1:100 at 37 °C overnight. An additional portion of trypsin was added and incubated for 4 h. Peptides were collected by centrifugation. The 200 µg peptides for the phosphoproteomics pipeline were digested a third time using trypsin.

Isobaric labelling was performed using Tandem Mass Tag (TMTpro 16plex) reagents (Thermo Fisher Scientific). The labelled samples were each combined into two pooled TMT sets, concentrated using vacuum centrifugation, before SDC was removed by acidification with 10% TFA and subsequent centrifugation. The peptide samples were purified using Pierce peptide desalting spin columns (ThermoFisher Scientific) according to the manufacturer's instructions.

The 200 µg peptide samples were subjected to sequential phosphopeptide enrichment using first High-Select TiO2 Phosphopeptide Enrichment Kit. Unbound peptides were then transferred to columns of the High-Select Fe-NTA Enrichment Kit (both ThermoFisher Scientific). The eluted phosphopeptide samples from both enrichments were pooled and fractionated into 13 fractions by increasing ACN concentration from 7% to 50% using the Pierce High pH Reversed-Phase Peptide Fractionation Kit (ThermoFisher Scientific).

#### 2.5.2 Nano-liquid chromatography tandem mass spectrometry (nLC-Ms/Ms)

The fractions of the whole phosphoproteome samples were analyzed on an Orbitrap Lumos™ Tribrid™ mass spectrometer interfaced with an Easy-nLC1200 liquid chromatography system (ThermoFisher Scientific). Peptides were trapped on an Acclaim Pepmap 100 C18 trap column (100 *μ*m × 2 cm, particle size 5 *μ*m, ThermoFisher Scientific) and separated on an in-house packed analytical column (75 *μ*m × 35 cm, particle size 3 *μ*m, Reprosil-Pur C18, Dr. Maisch). The fractions were separated using a linear gradient from 7% to 35% B over 47 min, 35% to 100% over 3 min, and 100% B for 10 min at a flow of 300 nl/min. Solvent A was 0.2% formic acid and solvent B was 80% acetonitrile, 0.2% formic acid.

MS scans were performed at a resolution of 120,000, and an m/z range of 375–1,500. MS/MS analysis was performed in a data-dependent, with top speed cycle of 3 s for the most intense doubly or multiply charged precursor ions. Precursor ions were isolated in the quadrupole with an isolation window of 0.7 m/z, with dynamic exclusion set to 10 ppm and duration of 45 s. Isolated precursor ions were fragmented by higher-energy collision dissociation (HCD) using a collision energy of 38%. MS2 spectra were detected in the Orbitrap with the fixed first m/z of 100 and a maximum injection time of 150 ms.

#### 2.5.3 Phosphoproteomic data analysis

The data files of the fractions from each set were merged for identification and relative quantification using Proteome Discoverer version 2.4 (ThermoFisher Scientific). Identification was performed using Mascot version 2.5.1 (Matrix Science) as a search engine by matching against the *Rattus* database of Uniprot (61,432 entries). The precursor mass tolerance was set to 5 ppm and fragment mass tolerance to 30 mmu. A stepped approach from zero to three missed cleavages was used for the phosphoproteome. Variable modifications of methionine oxidation; fixed cysteine alkylation, and TMTpro-label modifications of N-terminus and lysine were selected. Variable modification of serine, threonine or tyrosine phosphorylation was added. Percolator was used for the validation of identified phosphoproteins.

For quantification, TMTpro reporter ions were identified with 3 mmu mass tolerance in the MS2 HCD spectra for the phosphoproteome. The S/N values of the TMTpro reporters for each sample were normalized within Proteome Discoverer 2.4 on the total peptide amount. Only unique peptides, and those with a S/N ratio above 10, were considered for the protein quantification. Peptides were filtered for high confidence and resulting identified proteins for medium confidence.

#### 2.5.4 Phosphoproteomics: regulation analyses

The 9,103 quantified phosphopeptides were further processed in Perseus (14). Three valid values per sample group were required and expression values were log2 transformed allowing for statistical testing. Phosphopeptides were considered significantly regulated with a Welch's *t*-test *p* value of 0.05 and a fold change of at least 1.2 between IPC and NPC. For all phosphopeptides, Gene Ontology annotations derived from www.uniprot.org were imported, and enrichment analyses regarding specific GO terms (Molecular Function or Cellular Component) of the regulated phosphopeptides were performed within Perseus.

#### 2.5.5 Bioinformatic analysis

GO (Gene ontology) enrichment analysis of differentially expressed phosphoproteins using ShinyGo 0.76, a platform developed by South Dakota State University (15). The algorithm employed a fold enrichment approach based on the hypergeometric distribution, with subsequent false discovery rate (FDR) correction to ensure statistical reliability. To define the background gene set, all protein-coding genes in the rat genome were considered. The differentially expressed phosphoproteins, categorized as up- and downregulated, were then visualized using lollipop charts. These charts illustrated the fold enrichment for each enriched GO term in cellular components and molecular functions. For stringent significance assessment, a critical FDR threshold of <0.05 was applied in each GO enrichment analysis. Moreover, only GO terms with a minimum of 10 associated differentially expressed pathways were displayed. This criterion aimed to focus on biologically relevant enrichments while reducing noise in the results.

## 3 Statistical analysis

The statistical analyses were performed using IBM SPSS Statistics 27, GraphPad Prism 9 and R version 4.2.0. One-way ANOVA was used to compare physiological parameters between the different groups. The repeated measures of speckle tracking parameters were compared using a mixed-design ANOVA, which was adjusted using the Bonferroni correction. For the comparison of infarct size between the IPC-13.5 min group and NPC-13.5 min group an independent sample *t*-test was used. The level of statistical significance was set at *p* < 0.05.

## 4 Results

In both the first and second set of experiments, physiological parameters were similar in the different ischemia duration groups (Tables 1, 2). In the acute phase of the study, where myocardial infarction size was assessed and tissue biopsies were collected for phosphoproteomic analysis, all 26 rats successfully underwent the operative procedure without any instances of mortality. During the survival phase, which involved echocardiographic analysis, a total of 42 rats were operated on. Within this cohort, mortality was observed in two cases: two rats (representing 2 out of 8) from the non-preconditioned 13.5 min group and one rat (1 out of 7) from the non-preconditioned 12 min group.

**Table 1:** Physiological characteristics of rats exposed to different ischemia durations in the first set of experiments.

		Groups based on LAD ligation duration					
Variable	NPC 10 min	NPC 11 min	NPC 12 min	NPC 13.5 min	IPC 12 min	IPC 13.5 min	IPC 15 min
N	6	6	6	6	6	6	6
Age (weeks)	6.8 ± 0.4	6.9 ± 0.4	6.9 ± 0.6	7.0 ± 0.4	6.8 ± 0.6	7.2 ± 0.6	6.5 ± 0.4
Weight (g)	327 ± 23	330 ± 15	333 ± 40	327 ± 40	320 ± 28	305 ± 23	342 ± 10
Temperature (°C)	37.4 ± 0.17	37.3 ± 0.18	37.4 ± 0.19	37.5 ± 0.2	37.6 ± 0.15	37.5 ± 0.15	37.3 ± 0.1
Heart rate (bpm)	271 ± 7.5	259 ± 21	266 ± 11	281 ± 9	265 ± 12	277 ± 20	262 ± 19
ET- CO_2_ (%)	5.8 ± 0.4	5.6 ± 0.4	5.4 ± 0.3	5.5 ± 0.4	5.5 ± 0.3	5.1 ± 0.3	5.2 ± 0.6
O_2_ saturation (%)	98.8 ± 0.4	98.9 ± 0.3	98.8 ± 0.2	98.9 ± 0.3	98.2 ± 0.2	99.0 ± 0.2	98.9 ± 0.3

Data are shown as mean and standard deviation.

LAD, left anterior descending artery; IPC, ischemic preconditioning; ET, end-tidal; BPM, beat per minute; IPC, ischemic preconditioning; NPC, no ischemic preconditioning.

**Table 2:** Physiological characteristics of rats exposed to either ischemic preconditioning or not in the infarction-size experiments.

Variable	Group	
	IPC-13.5 min	NPC-13.5 min
N	5	5
Age (weeks)	6.7 ± 0.5	7.1 ± 0.3
Weight (g)	312 ± 18	322 ± 22
Temperature (°C)	37.4 ± 0.2	37.4 ± 0.13
Heart rate (bpm)	283 ± 21	290 ± 28
ET- CO_2_ (%)	5.2 ± 0.3	5.6 ± 0.4
O_2_ saturation (%)	98.8 ± 0.3	99.0 ± 0.4
AAR/LV area (%)	39.2 ± 2.5	42.1 ± 1.9

Data are shown as mean and standard deviation.

IPC, ischemic preconditioning; ET, end-tidal; BPM, beat per minute; AAR, area at risk; LV, left ventricle; NPC, no ischemic preconditioning.

### 4.1 Impact of IPC on the incidence and severity of cardiac dysfunction at different durations of ischemia

The incidence of reversible myocardial akinesia (akinesia at 4 h reperfusion that resolved by 48 h after reperfusion), and the median WMSI is presented in Figure 2, akinesia is defined as an WMSI of 3. Among rats without IPC, none developed akinesia at 4 h when the duration of ischemia was shorter than 11 min. When the duration of ischemia was 11 min, 17% developed akinesia at 4 h that resolved by 48 h, and 50% developed akinesia at 4 h that resolved by 50%. At ischemia duration of 12 min, 66% developed akinesia at 4 h, and none resolved completely by 48 h. There was no duration of ischemia that consistently induced reversible akinesia, i.e., akinesia present at 4 h that had resolved at 48 h in rats without IPC (Figures 2C,E**)**. In contrast, among rats with IPC, ischemia durations between 13.5 and 15 min induced reversible akinesia in 83% and 66% of rats respectively (Figures 2B,D).

**Figure 4:**
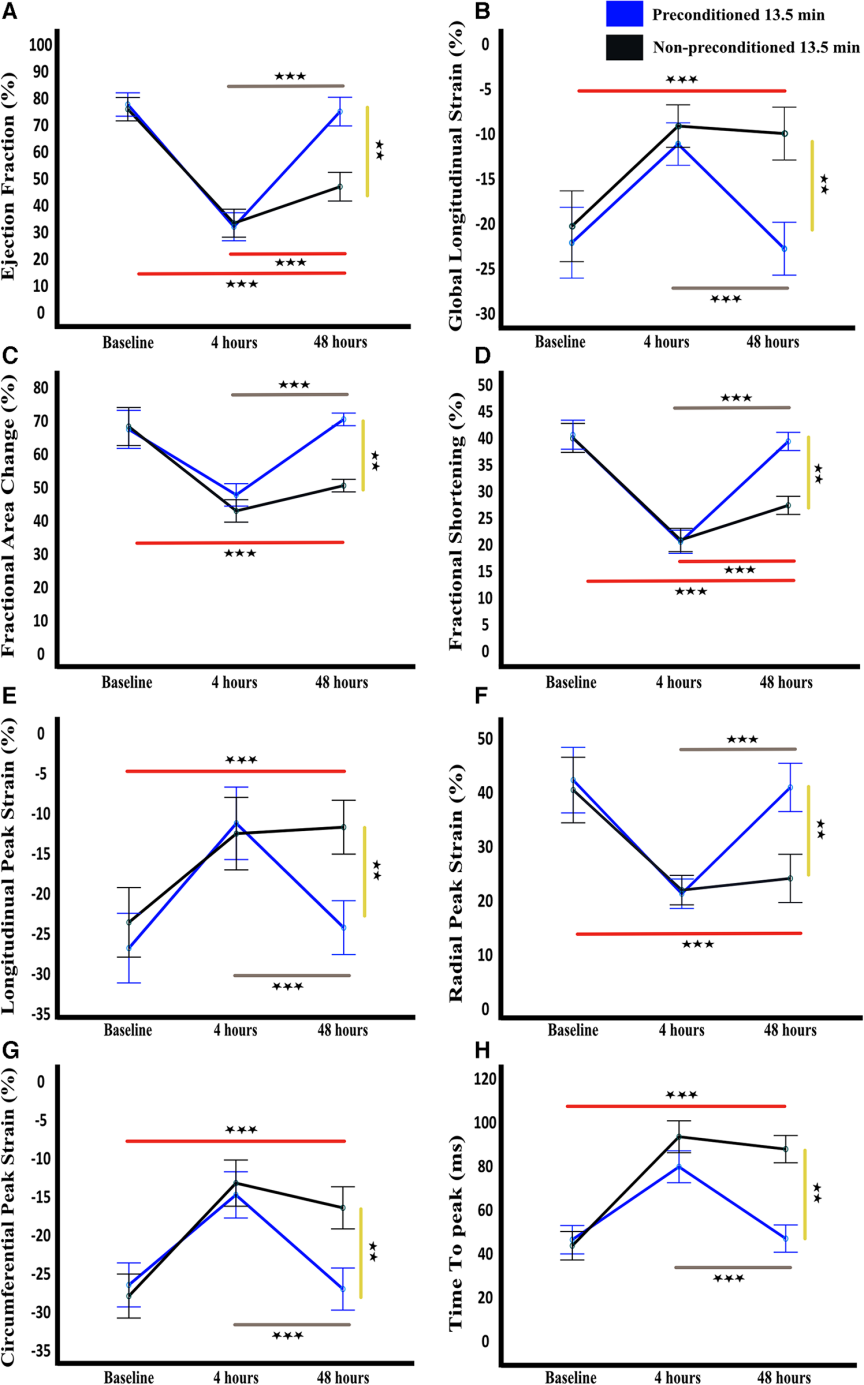
Temporal functional and strain cardiac parameters. Ejection fraction (**A**), global longitudinal strain (**B**), fractional area change (**C**), fractional shortening (**D**), longitudinal peak strain (**E**), radial peak strain (**F**), circumferential peak strain (**G**), and time to peak (**H**) are shown as mean and standard deviation at baseline, 4 h, and 48 h after LAD reperfusion. Statistical analysis was performed using mixed-design ANOVA with the Bonferroni correction, which revealed a significant decline in all parameters at 4 h in both groups as compared to baseline. However, at 48 h, a significant improvement was observed in all parameters in the preconditioned group, indicating recovery, whereas no significant improvement was observed in the non-preconditioned group. The grey and red lines indicate significance across time points for the preconditioned-13.5 min and non-preconditioned-13.5 min groups, respectively. The yellow line denotes significance between the two groups at 72 h. *n* = 6/group. ** = *p* < 0.01, *** = *p* < 0.001.

Other indices of global and regional cardiac function paralleled the WMSI of the affected segment, with transient worsening with full recovery in rats with but not without IPC (Figures 2D,E, 3–4, Table 3).

**Table 3:** Quantitative measurements of cardiac function parameters using speckle tracking analysis, presented as mean **± **SD.

Variable	Groups (13.5 min of LAD ligation)	Baseline	4 h after LAD reperfusion	48 h after LAD reperfusion	*P* value between 4 h and 48 h
Ejection fraction (%)	IPC	77.3% ± 3.8	31.6% ± 4.4	74.6 ± 4.6	<0.001
	NPC	75.6 ± 5.6	37.0 ± 6.7	45.1 ± 6.9	=0.07
Global longitudinal strain (%)	IPC	−22.2 ± 3.6	−11.2 ± 2.3	−22.8 ± 4.2	<0.001
	NPC	−20.3 ± 4.9	−9.2 ± 2.8	−10.3 ± 1.7	=0.4
Fractional shortening (%)	IPC	40.2 ± 2.7	20.7 ± 2.1	39.5 ± 1.8	<0.001
	NPC	39.3 ± 2.2	22.0 ± 3.2	25.5 ± 2.4	=0.06
Fractional area change (%)	IPC	66.8 ± 5.0	47.2 ± 4.6	69.8 ± 1.9	<0.001
	NPC	67.3 ± 7.3	44.1 ± 3.3	48. ± 3.1	=0.06
Time to peak (ms)	IPC	49.0 ± 4.4	81.3 ± 5.8	49.5 ± 6.1	<0.001
	NPC	46.3 ± 8.7	94.7 ± 9.2	89.3 ± 7.2	=0.28
Circumferential peak strain (%)	IPC	−26.7 ± 2.5	−15.5 ± 2.3	−27.3 ± 3.3	<0.001
	NPC	−28.2 ± 3.6	−13.5 ± 3.5	−16.7 ± 2.8	=0.11
Longitudinal peak strain (%)	IPC	−28.0 ± 5.2	−11.8 ± 3.6	−25.3 ± 4.5	<0.001
	NPC	−24.7 ± 4.5	−13.2 ± 6.3	−12.3 ± 3.1	=0.76
Radial peak strain (%)	IPC	27.3 ± 1.1	11.1 ± 2.3	30.6 ± 2.2	<0.001
	NPC	25.1 ± 3.2	12 ± 2.8	15.9 ± 3.1	=0.06

LAD, left anterior descending artery; IPC, ischemic preconditioning; NPC, non-ischemic preconditioning; ms, milliseconds.

### 4.2 Infarction size

**Figure 5:**
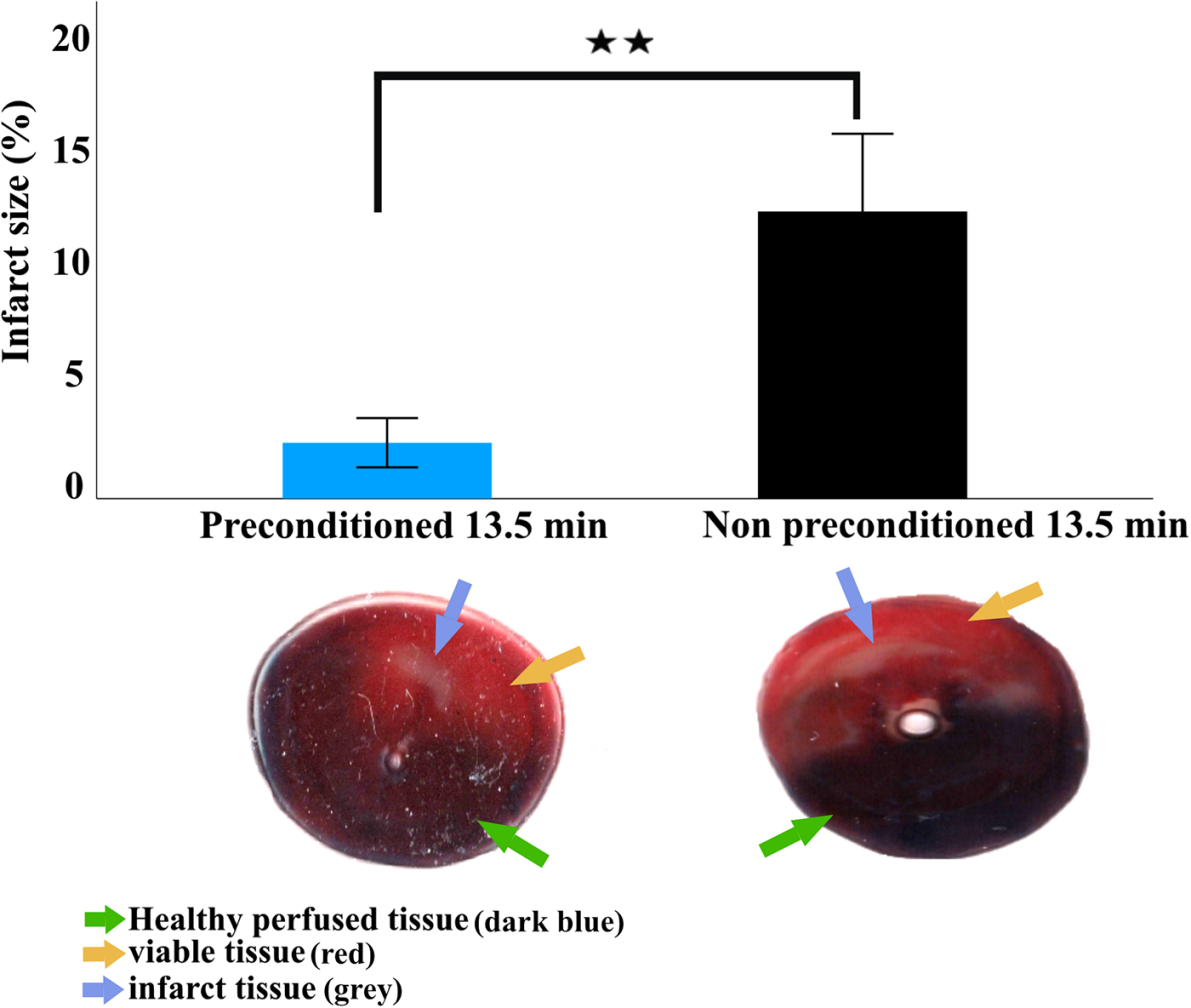
Infarct size 2 h after LAD reperfusion. Infarction size is expressed as mean and standard deviation. Statistical analysis was performed using an independent sample *t*-test. Representative images of transversely sectioned Evans blue perfused and TTC-stained hearts showing live tissue (yellow arrow), infarcted tissue (blue circles), and healthy perfused tissue (green arrow). The viable tissue represents the non-infarct area within the area at risk while the healthy perfused tissue represent the area of the heart not supplied by the ligated artery. The area at risk is defined as the sum of the live tissue and infarcted tissue. ** = *p* < 0.01, *n* = 5 per group.

As assessed by Evans blue/TTC double staining IPC-13.5 min group had significantly lower infarction size (1.8% ± 1.1%) compared to the NPC-13.5 min group (12.8% ± 3.3; *p* < 0.005; Figure 5).

### 4.3 Phosphoproteomic analysis

**Figure 6:**
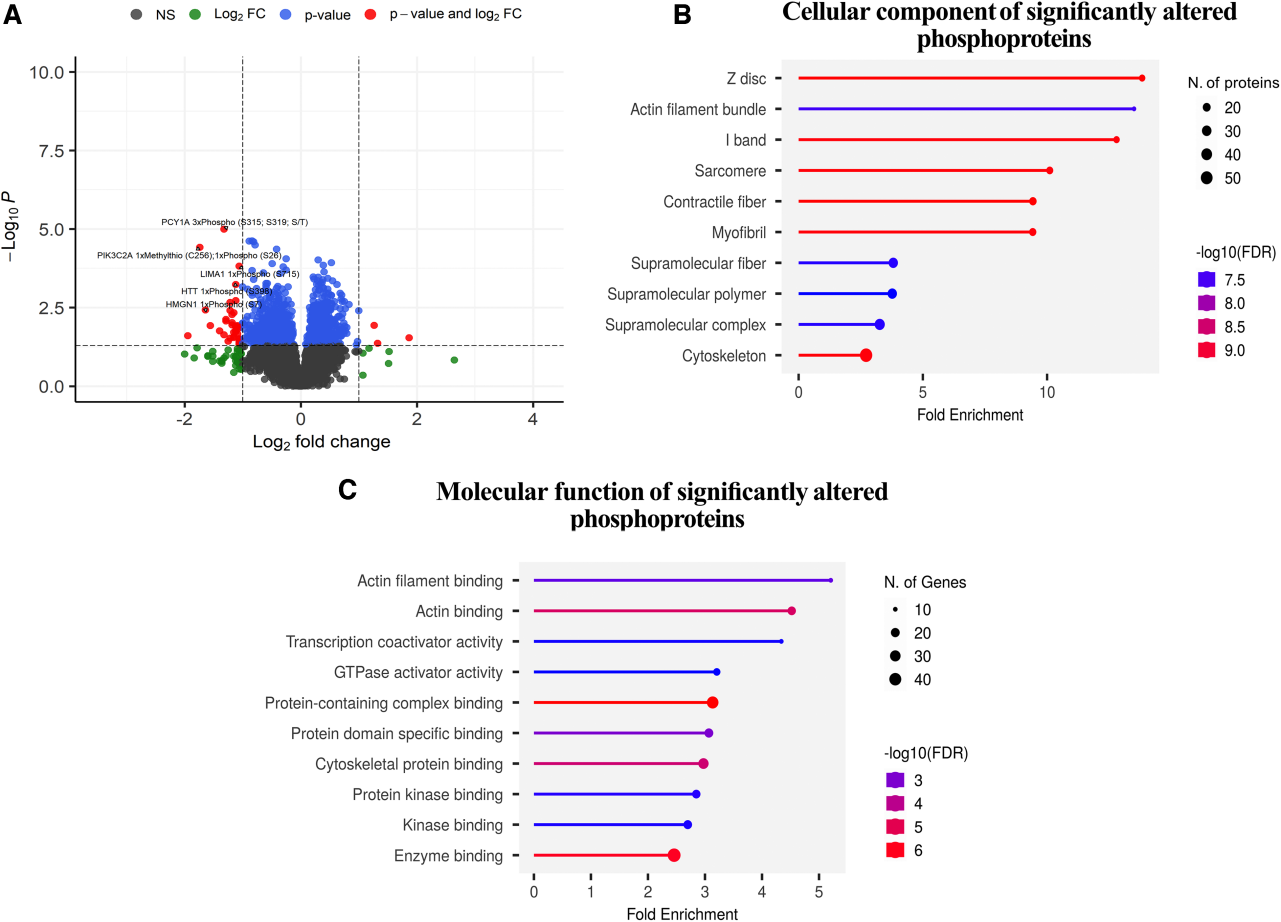
Phosphoproteomic analysis. (**A**), Volcano plot depicting the significant phosphopeptides in terms of their significance levels and fold changes in expression. Phosphopeptides with *p*-values less than 0.05 were considered to be statistically significant (red and blue points in the plot). Of these, those with more than a two-fold expression are additionally demarcated (phosphopeptides denoted in red). The top 5 upregulated and downregulated phosphopeptides with the highest changes in expression are labelled. The phosphopeptides represented in black (NS) and green (log2FC) points denote the ones that were not statistically significant and were not considered for downstream analyses. (**B,C**), Gene Ontology term enrichment analysis of differentially expressed phosphoproteins in preconditioned compared to non-preconditioned animals based on cellular components and molecular function.

A total of 9,103 phosphopeptides from 2,658 proteins were identified, with phosphorylation states ranging from mono- to pentaphosphorylated. Specifically, we observed 6,609 monophosphorylated, 1,412 diphosphorylated, 276 triphosphorylated, 45 tetraphosphorylated, and 5 pentaphosphorylated phosphopeptides. Among these, 786 phosphopeptides displayed differential regulation. In the comparison between the IPC group and the NPC group, 412 phosphopeptides were upregulated, and 374 were downregulated based on an absolute log2 fold change (log2FC) > 0.26 and a *P*-value < 5% in Welch's *t*-test. The distribution of these altered phosphopeptides was as follows: 565 monophosphorylated, 119 diphosphorylated, 30 triphosphorylated, 4 tetraphosphorylated, and 1 pentaphosphorylated. The primary residues phosphorylated were serine, threonine, and tyrosine (Figure 6A and Supplementary Tables S1, S2).

### 4.4 Functional enrichment

Figure 6 and Supplementary Table S3 show the results of the functional enrichment analysis, which related the differentially regulated phosphopeptides to specific subcellular localizations (Figure 6B) and molecular functions (Figure 6C). Cellular component analysis: The most significantly enriched cellular component in the differentially phosphorylated proteins is the Z-disc, with a fold enrichment value of 13.824169 and an enrichment FDR of 3.46E-10. The phosphorylated proteins involved in this pathway include myopalladin (MYPN), filamin-C (FLNC), alpha-crystallin B chain (CRYAB), ankyrin 2 (ANK), myozenin 2 (MYOZ2), among others. Actin filament bundle and I-band also show significant enrichment with fold enrichment values of 13.49972421 and 12.7947096, respectively. Components like sarcomere, contractile fiber, and myofibril have similar significant enrichment but with slightly reduced fold enrichment values ranging from 9 to 10. The cytoskeleton has the highest number of phosphorylated proteins involved, with 56 genes but has a fold enrichment of 2.716437499 (Figure 6A and Supplementary Table S3).

Molecular Function Analysis: The top enriched molecular function in the differentially phosphorylated proteins is actin filament binding with a fold enrichment value of 5.206505207 and an enrichment FDR of 0.00207426. The phosphorylated proteins involved in this function are catenin alpha 1 (CTNNA1), ITPR-interacting domain-containing 2 (ITPRID2), myosin-7 (MYH7), and supervillin (SVIL), among others. Actin binding is another significantly enriched function with a fold enrichment of 4.521438732. Transcription coactivator activity has a fold enrichment of 4.338754339, and GTPase activator activity has 3.207207207. Enzyme binding involves the highest number of phosphorylated proteins, with 48 phosphorylated proteins, but its fold enrichment is ‘2.45888618, indicating that while many phosphorylated proteins have this function, its enrichment in the differentially phosphorylated proteins is comparatively lower (Figure 6B and Supplementary Table S3).

## 5 Discussion

The most important findings of this study are that (i) across different non-necrotic and necrotic durations of ischemia, transient myocardial stunning was more often observed to a greater extent with than without IPC; and (ii) the majority of the more pronounced phosphoproteomic changes induced by IPC affected the contractile apparatus. Taken together, these findings suggest that IPC accentuate myocardial stunning in the setting of ischemia-reperfusion; and that myocardial stunning may at least be involved in the cardioprotective effect of IPC.

The current understanding of myocardial stunning is incomplete and previous studies that hypothesized that myocardial stunning is a deleterious process and have attempted to prevent or reduce myocardial stunning by IPC or other interventions have not succeeded to do so (3, 5, 6, 16). In our study, IPC accentuated rather than prevented myocardial stunning; with fully reversible akinesia after a subsequent more prolonged ischemic insult consistently observed only with IPC.

Reversible akinesia (stunning) after prolonged ischemia was rare among rats that were not subjected to ischemic preconditioning. In this group of rats, 10 min of ischemia did not result in post-ischemic akinesia in any rats, whereas ischemia of 11 min or greater resulted in irreversible cardiac dysfunction in the majority of the rats. Conversely, in the group that were subjected to ischemic preconditioning, ischemia durations between 13.5 to 15 min resulted in fully reversible akinesia in almost all rats. Group differences in the changes over time in other indices of global and regional myocardial function mimicked those observed for akinesia and wall motion score index, further supporting the notion that IPC supported post-ischemic reversible depression of cardiac function (17–19). In addition to increasing the incidence of reversible post-ischemic akinesia (stunning), preconditioning significantly reduced infarct size. Hence, IPC appears to both associate with increased propensity and extent for myocardial stunning and reduce infarct size.

Our experiments do not establish whether the greater propensity and extent for myocardial stunning and the reduction in infarct size after preconditioning are causally linked. However, our phosphoproteomic analysis showed that the majority of the phosphosites that were altered by IPC were related to myocardial contraction, suggesting that IPC exerts part of cardioprotective effects by affecting contractile activities.

Specifically, these altered phosphosites included sarcomere proteins, Z-disc, I-band, and contractile fibers associated with actin binding. Subcellular localization analysis indicated that the altered phosphoproteins were enriched in the Z disc, I band, sarcomere, contractile fiber, myofibril, and cytoskeleton. These subcellular structures are essential components of the cardiac sarcomere and are critical for maintaining contractile function (20–23). Functional enrichment analysis of the significantly changed phosphoproteins identified actin binding as particularly affected, further suggesting that the observed changes may influence contractile function during ischemia-reperfusion injury (24, 25).

These phosphoproteomic results suggests that IPC-induced changes in the phosphorylation status of contractile proteins may explain the greater propensity for myocardial stunning in the IPC group. The orchestration of left ventricular contractile function is a result of intricate molecular interplays involving numerous proteins. The significance of Myopalladin (MYPN) has been highlighted by its role in linking sarcomeric thin filaments to the intercalated disc, ensuring structural integrity during muscle contractions. Myopalladin knockout mice develop cardiac dilation and show a maladaptive response to mechanical pressure overload (26). Filamin-C (FLNC) maintains cardiac muscle structural integrity due to its actin-binding capabilities; mutations in this protein are often tied to cardiomyopathies. Filamin C truncation mutations are associated with arrhythmogenic dilated cardiomyopathy and changes in the cell-cell adhesion structures (27). Catenin Alpha 1 (CTNNA1) is integral to the cadherin-catenin complex, crucial for cell-cell adhesion, with disruptions in its function or phosphorylation potentially leading to cardiomyocyte detachment and unique forms of cardiomyopathy and increased vulnerability to fatality after cardiac stress (28). Alpha-E-catenin inactivation disrupts the cardiomyocyte adherens junction, resulting in cardiomyopathy and susceptibility to wall rupture (28). The role of ITPR-interacting domain-containing 2 (ITPRID2) remains relatively less explored in the context of cardiac function, though it holds potential in modulating intracellular calcium signalling, a vital aspect of cardiac contractility. KRAS-induced actin-interacting protein regulates inositol 1, 4, 5-trisphosphate-receptor-mediatedcalcium release (29). Calsarcin-1 protects against angiotensin-II induced cardiac hypertrophy (30). Myosin-7 (MYH7) encodes the beta-myosin heavy chain, a vital component of the sarcomeric apparatus, and its mutations are extensively linked to cardiomyopathies (31). Supervillain (SV2) is involved in myofibrillar assembly and localizes at the ends of myotubes. Archvillin, a muscle-specific isoform of supervillin, is an early expressed component of the costameric membrane skeleton (32). Knockdown of archvillin by siRNA inhibits myofibril assembly in cultured skeletal myoblast (33). It interacts with critical cardiac and skeletal muscle cell structures like costameres and sarcomeres, partnering with proteins like nebulin, dystrophin, and *γ*-sarcoglycan. Mutations in these proteins are linked to significant muscular disorders, with nebulin mutations associated with congenital nemaline myopathy. Congenital myopathies and others like dystrophin causing muscle disorders that can affect both cardiac and skeletal systems (34). Spectrum of muscular dystrophies associated with sarcolemmal-protein genetic defects (35). Costamere proteins and their involvement in myopathic processes (36). Proteins like CRYAB, ANK2, and MYOZ2 play crucial roles in cardiac health. CRYAB modulates cardiomyocyte apoptosis in cardiac disorders, ANK2 regulates cardiac ion channels, and MYOZ2 mutations are linked to hypertrophic cardiomyopathy. Role of α-crystallin B as a regulatory switch in modulating cardiomyocyte apoptosis by mitochondria or endoplasmic reticulum during cardiac hypertrophy and myocardial infarction (37). Indeed, phosphorylation of contractile proteins is known to regulate cardiac contractility and may play a central role in stunning and recovery processes (38–40).

In conclusion, our study show that IPC induces changes in phosphosites of proteins involved in myocardial contraction; and both accentuates post-ischemic myocardial stunning and reduces infarct size.

## 6 Limitations

This study has limitations. First, myocardial stunning may involve other aspects of cardiac function than what could be examined in this study. However, we used sophisticated echocardiographic equipment dedicated for small animals to examine several established indices of regional and global cardiac function. Second, the experimental groups were relatively small, and we studied only 5 different durations of ischemia. It is possible that the incidence of reversible post-ischemic akinesia would have been different at other durations of ischemia. However, we showed that within a range of 1 min, rats that were not exposed to IPC either did not develop post-ischemic akinesia (10 min) or developed irreversible dysfunction (11 min); whereas in rats that were exposed to IPC, almost all rats that were exposed to ischemia durations between 13.5 to 15 min developed reversible akinesia. Hence, the differences between the groups in the incidence of reversible post-ischemic akinesia are clear. Although regression analysis could potentially offer valuable insights into the effects of ischemic preconditioning on cardiac parameters and phosphosite changes, the small sample size of our study poses significant challenges. Specifically, it increases the risk of overfitting the model and diminishes statistical power, thereby impacting the reliability of any inferred relationships. Third, we studied only male rats. The extent to whether our findings are consistent across sex should be examined. Lastly, while the phosphoproteomic alterations induced by IPC are consistent with the notion that IPC and myocardial stunning are intertwined, the functional relevance of individual phosphorylation events requires further investigation using targeted experiments.

## Data Availability

The mass spectometry proteomics data have been deposited in the ProteomeXchange Consortium via the PRIDE (41) partner repository, with the dataset identifier PXD050441.
