# Supplementary material for: Ischemic preconditioning affects phosphosites and accentuates myocardial stunning while reducing infarction size in rats
Source: Front Cardiovasc Med. 2024 Mar 15;11:1376367. doi: 10.3389/fcvm.2024.1376367 (PMC10978780; doi:10.3389/fcvm.2024.1376367)
Supplement: Supplementary file 1 [file Table1.docx]

**Supplementary Table S1:** Upregulated phosphopeptides in the preconditioned group relative to the non-preconditioned group.

| **Protein Names** | **Modifications in Master Proteins** | **Log2FC** | **-Log Welch's T-test p-value** |
| --- | --- | --- | --- |
| Myomesin 2 | A0A8I6AC90 1xPhospho [S1119(100)] | 0.331206 | 1.681736925 |
| Titin;Cardiac titin N2B isoform | A0A8I5ZUN3 1xPhospho [S3870(100)];Q7TMZ9 1xPhospho [S354(100)] | 0.343954 | 2.533559629 |
| Histidine rich calcium binding protein | A0A8I6AUL9 1xPhospho [S538(100)] | 0.344446 | 1.335724745 |
| Plectin 11 | Q6S395 1xPhospho [S4247(99.3)] | 0.338416 | 1.57108559 |
| Centrosomal protein 131 | D4AEL8 1xPhospho [S304(100)] | 0.330149 | 1.462176141 |
| Nascent polypeptide associated complex subunit alpha | M0R9L0 1xPhospho [S1422(100)] | 0.354665 | 2.477864653 |
| Stathmin | A0A8I5ZX99 1xPhospho [S37(100)] | 0.330645 | 1.749849254 |
| Microtubule-associated protein 1B | A0A8I5ZWW4 2xPhospho [S1786(100);T1788(100)] | 0.334419 | 1.331701727 |
| G-protein signaling modulator 1 | A0A8I5ZZF6 1xPhospho [S489(100)] | 0.333737 | 1.648442278 |
| Nascent polypeptide associated complex subunit alpha | M0R9L0 2xPhospho [S942(100);S/T] | 0.369234 | 1.583460346 |
| AHNAK nucleoprotein; AHNAK nucleoprotein | A0A0G2JU96 1xPhospho [S5283(100)];A0A0G2JUA5 1xPhospho [S5411(100)] | 0.334867 | 1.882542006 |
| Oxidation resistance protein 1 | Q4V8B0 2xPhospho [S194(99.1);T/S] | 0.314873 | 1.693822176 |
| DNA-directed RNA polymerase subunit | A0A8I6ASE2 2xPhospho [S1899(96.9);S1906(100)];2xPhospho [S1913(96.9);S1920(100)] | 0.35311 | 1.320163694 |
| Smoothelin; Smoothelin | A0A8I5ZNI1 1xPhospho [S708(99.2)];A0A8I6A098 1xPhospho [S385(99.2)] | 0.335184 | 2.349848312 |
| Sarcalumenin | A0A8I6G9C9 1xPhospho [S316(99.6)] | 0.343954 | 2.059107058 |
| AHNAK nucleoprotein; AHNAK nucleoprotein | A0A0G2JU96 1xPhospho [S94(100)];A0A0G2JUA5 1xPhospho [S94(100)] | 0.335603 | 1.444818707 |
| GRB10 interacting GYF protein 1 | D3ZQJ3 1xPhospho [S404(100)] | 0.36257 | 2.370208456 |
| Serine/arginine-rich splicing factor 1 | A0A8I6GMR8 1xPhospho [S199(100)] | 0.333126 | 1.409036961 |
| Translocase of outer mitochondrial membrane 34 | A0A8I6AAW4 1xPhospho [S186(100)] | 0.352672 | 1.892006191 |
| Protocadherin 12 | F1MA46 1xPhospho [S1142(100)] | 0.358048 | 2.588993681 |
| Serine/arginine repetitive matrix 2 | A0A8I6A0A2 4xPhospho [S1433(100);S1435(100);S1437(100);S1438(100)] | 0.334867 | 2.319683879 |
| Stromal interaction molecule 1 | P84903 2xPhospho [S519(100);S521(100)] | 0.357157 | 1.598094293 |
| Striated muscle enriched protein kinase | A0A8I6ADG2 1xPhospho [S1177(100)] | 0.366428 | 1.582641164 |
| Cask-interacting protein 2 | D4A9T0 1xPhospho [S396(100)] | 0.354431 | 1.972311784 |
| PPARGC1 and ESRR induced regulator, muscle 1 | D3ZH76 1xPhospho [S632(99)] | 0.353637 | 3.583063009 |
| Phosphodiesterase | A0A8I6AT13 1xPhospho [S182(100)] | 0.356935 | 1.721465886 |
| Non-specific serine/threonine protein kinase | A0A0G2JUP3 1xPhospho [S135(100)] | 0.36257 | 1.311533122 |
| Alpha-kinase 3 | A0A8I5ZR19 1xPhospho [S216(100)] | 0.34192 | 1.510755261 |
| Microtubule-associated protein | A0A8I5ZSY4 1xPhospho [S1875(100)] | 0.349008 | 1.502962939 |
| Histidine rich calcium binding protein | A0A8I6AUL9 1xPhospho [S342(100)] | 0.355659 | 1.310031128 |
| Leucine rich repeats and calponin homology domain containing 3 | A0A8I6B669 1xPhospho [S194(100)] | 0.354283 | 1.389807509 |
| Sorbin and SH3 domain containing 2 | A0A8I6AA99 1xPhospho [S51(100)] | 0.36257 | 1.950150586 |
| Synaptopodin 2-like | D3ZZ68 2xPhospho [T705(100);T713(100)] | 0.364572 | 2.253543116 |
| Ankyrin-3 | A0A0G2K4D0 1xPhospho [S1412(100)] | 0.36059 | 1.407599689 |
| Dystonin; Dystonin | A0A8I6ABJ6 1xPhospho [S1998(100)];A0A8I5ZQD4 1xPhospho [S4056(100)] | 0.351985 | 1.716503322 |
| ADP-ribosylhydrolase like 1 | A0A8J8XAN4 1xPhospho [S913(100)] | 0.359542 | 1.978067603 |
| F-box protein 38 | D3ZIK8 1xPhospho [S742(100)] | 0.377638 | 1.682844678 |
| 55 kDa erythrocyte membrane protein | Q5BK33 1xPhospho [S57(100)] | 0.368387 | 1.314697055 |
| Titin; Titin | A0A8I5ZUN3 1xPhospho [S1389(99.2)];A0A8I6A794 1xPhospho [S1344(99.2)] | 0.369234 | 2.137455628 |
| Tight junction protein 2 | Q3ZB99 1xPhospho [S107(100)] | 0.384143 | 1.467242021 |
| Superoxide dismutase [Cu-Zn] | Q6LDS4 1xPhospho [S106(100)] | 0.371256 | 2.057239996 |
| Complement component C1q receptor | Q9ET61 1xPhospho [S636(97.8)] | 0.378512 | 1.441977715 |
| Coiled-coil domain-containing protein 91 | Q6AY97 1xPhospho [S46(100)] | 0.383329 | 2.728025188 |
| Misshapen-like kinase 1 | A0A8I6B5X1 1xPhospho [S768(100)] | 0.391425 | 1.443872471 |
| Propionyl-CoA carboxylase alpha chain, mitochondrial | A0A8I5ZL37 1xPhospho [S240(99.6)] | 0.359896 | 2.793290528 |
| Spectrin, alpha, non-erythrocytic 1;  Spectrin, alpha, non-erythrocytic 1 | A0A0G2JTH0 1xPhospho [S2235(100)];A0A0G2K1Y8 1xPhospho [S2255(100)] | 0.361099 | 1.349351037 |
| Dystrophin | A0A8I6GKZ8 1xPhospho [S3603(100)] | 0.38109 | 2.888621452 |
| PTPRF interacting protein alpha 1 | D3ZZ81 1xPhospho [S696(99.1)] | 0.388271 | 1.613285768 |
| Furry-like protein | C0IXW6 1xPhospho [S1954(99.3)] | 0.368387 | 1.347454687 |
| Carbonic anhydrase 2 | P27139 1xPhospho [S87(100)] | 0.373458 | 1.510435609 |
| Zfr protein | B1WC00 1xPhospho [S108(100)] | 0.379848 | 1.591889428 |
| Microtubule-associated protein 1A | G3V7U2 1xPhospho [S1206(100)] | 0.351472 | 1.318865965 |
| Xin actin-binding repeat containing 1 | A0A8I5YBK9 1xPhospho [T766(100)] | 0.347923 | 1.528628788 |
| RIKEN cDNA 8030462N17 gene | D4A3X1 1xPhospho [S66(100)] | 0.372809 | 1.677534239 |
| Plakophilin-2 | A0A8I6A597 1xPhospho [S555(100)] | 0.375867 | 1.330738072 |
| Histidine rich calcium binding protein | A0A8I6AUL9 1xPhospho [S656(100)] | 0.385025 | 1.696162275 |
| Zinc finger protein 646 | A0A0G2KA68 1xPhospho [S601(100)] | 0.366128 | 2.246548746 |
| Caldesmon 1 | A0A8I6GCL3 1xPhospho [S477(99.1)] | 0.38529 | 1.362033824 |
| Myosin XVIIIa | A0A8I6A163 1xPhospho [S2246(100)] | 0.380822 | 1.871670615 |
| RAF proto-oncogene serine/threonine-protein kinase | P11345 1xPhospho [S43(100)] | 0.383329 | 1.337786525 |
| Hemoglobin alpha, adult chain 1; Globin c2 | A0A0A0MP82 1xPhospho [S50(100)];1xPhospho [S95(100)];A0A1K0FUH3 1xPhospho [S50(100)] | 0.39689 | 1.352531068 |
| ArfGAP with SH3 domain, ankyrin repeat and PH domain 1 | A0A8I5ZYZ1 1xPhospho [S963(100)] | 0.398549 | 1.365586817 |
| Protein phosphatase 6, regulatory subunit 2 | A0A0G2JV49 1xPhospho [S696(99.5)] | 0.410088 | 1.833880337 |
| Beta-glo; Hemoglobin, beta adult major chain; Hemoglobin subunit beta-1; Hemoglobin, beta adult major chain | Q6PDU6 1xPhospho [S140(100)];A0A0G2JSW3 1xPhospho [S140(100)];P02091 1xPhospho [S140(100)];A0A8I6AUL4 1xPhospho [S140(100)] | 0.407658 | 2.957280087 |
| LIM domain binding 3; LIM domain binding 3 | A0A0G2JXR0 1xPhospho [S182(100)];A0A8I5Y5H4 1xPhospho [S227(100)] | 0.415037 | 1.776317845 |
| Eukaryotic translation elongation factor 1 delta | A0A8I5ZUU1 1xPhospho [S47(100)] | 0.400538 | 1.903349886 |
| Golgi brefeldin A resistant guanine nucleotide exchange factor 1 | F1M8X9 2xPhospho [S1779(100);S1780(100)] | 0.402098 | 1.824065594 |
| Sorbin and SH3 domain containing 1; Sorbin and SH3 domain containing 1; Sorbin and SH3 domain containing 1 | F1M865 1xPhospho [S236(100)];F1M866 1xPhospho [S601(100)];F1M8Z8 1xPhospho [S329(100)] | 0.399931 | 1.683514472 |
| Ubiquitin carboxyl-terminal hydrolase | A0A8I5ZV90 1xPhospho [S80(99.4)] | 0.402098 | 1.511633054 |
| E3 ubiquitin-protein ligase | D3ZLS5 1xPhospho [S1571(99.4)] | 0.393145 | 1.638608514 |
| LIM and calponin homology domains 1 | F1M392 1xPhospho [S994(100)] | 0.393145 | 1.731013768 |
| Catenin alpha 1 | Q5U302 1xPhospho [S692(100)] | 0.40632 | 1.600053786 |
| Nebulette | A0A0G2JWS2 1xPhospho [S833(100)] | 0.397071 | 1.671465551 |
| RCR-type E3 ubiquitin transferase | A0A8I6A2N3 1xPhospho [T1622(99.6)] | 0.405992 | 2.195779956 |
| Sorbin and SH3 domain containing 1; Sorbin and SH3 domain containing 1; Sorbin and SH3 domain containing 1 | F1M865 2xPhospho [S85(98.5);S86(98.5)];F1M866 2xPhospho [S84(98.5);S85(98.5)];F1M8Z8 2xPhospho [S54(98.5);S55(98.5)] | 0.402098 | 1.394857695 |
| Clustered mitochondria protein homolog | D3ZKG9 1xPhospho [S1295(100)] | 0.376854 | 1.311216785 |
| La ribonucleoprotein 1, translational regulator | A0A8I6GJC3 1xPhospho [T627(100)] | 0.387926 | 2.031896907 |
| INO80 complex subunit D | D3ZTM2 1xPhospho [S354(100)] | 0.398084 | 1.980996481 |
| A-kinase anchoring protein 9 | F1LPB4 1xPhospho [S3669(100)] | 0.39486 | 1.551006252 |
| A-kinase anchoring protein 2;A-kinase anchoring protein 2 | A0A8I6AAB5 1xPhospho [S318(100)];A0A8I5Y1X4 1xPhospho [S551(100)] | 0.386183 | 1.396823227 |
| Nebulette; Nebulette | A0A8I6B580 2xPhospho [S806(100);S810(100)];A0A0G2JWS2 2xPhospho [S899(100);S903(100)] | 0.393145 | 1.515513558 |
| Histidine rich calcium binding protein | A0A8I6AUL9 1xPhospho [S342(100)] | 0.384341 | 2.098535696 |
| Microtubule-associated protein 1A | G3V7U2 1xPhospho [S598(100)] | 0.376563 | 1.482917409 |
| A-kinase anchoring protein 6; A-kinase anchor protein 6 | G3V6M0 1xPhospho [S2196(99.2)];Q9WVC7 1xPhospho [S2195(99.2)] | 0.381871 | 2.746249327 |
| Myosin XVIIIa | A0A8I6A163 1xPhospho [S2334(100)] | 0.393481 | 1.79782177 |
| Mapk-regulated corepressor-interacting protein 1 | B0BN72 2xPhospho [S18(98.2);S/T] | 0.398549 | 1.473323366 |
| ATP-dependent RNA helicase | A0A0G2K4S4 1xPhospho [S480(100)] | 0.389567 | 1.917465319 |
| Microtubule-associated protein 1B | A0A8I5ZWW4 1xPhospho [T1626(100)] | 0.397964 | 3.178352458 |
| Filamin A | A0A8I5Y6H8 1xPhospho [S2319(99.1)] | 0.415037 | 1.820709719 |
| Ral GEF with PH domain and SH3 binding motif 2 | A0A8I6GHG1 1xPhospho [S329(100)] | 0.415037 | 1.407213565 |
| Adducin 1; Adducin 1 | A0A8I5Y1E2 1xPhospho [S12(99.6)];D3ZZ99 1xPhospho [S12(99.6)] | 0.420332 | 2.394583317 |
| Potassium voltage-gated channel subfamily H member 2 | O08962 1xPhospho [S322(100)] | 0.418501 | 4.356283817 |
| LIM domain 7; LIM domain 7 | A0A8I6AAI2 1xPhospho [S1567(100)];A0A8I5ZR48 1xPhospho [S1266(100)] | 0.419084 | 1.566217456 |
| Histidine rich calcium binding protein | A0A8I6AUL9 1xPhospho [S152(100)] | 0.40691 | 1.514381521 |
| Histidine rich calcium binding protein | A0A8I6AUL9 1xPhospho [S495(99.7)] | 0.426625 | 2.617318914 |
| Catenin alpha 3 | A0A0G2JX81 1xPhospho [T377(100)] | 0.432959 | 1.683814742 |
| Palmdelphin | Q4KM62 1xPhospho [S365(100)] | 0.415037 | 1.396511411 |
| BCAS3, microtubule associated cell migration factor | A0A8I6A420 2xPhospho [S480(98.2);S] | 0.415037 | 2.400738268 |
| Pseudopodium-enriched atypical kinase 1 | D4A563 1xPhospho [S568(98.7)] | 0.429988 | 2.381496922 |
| Tumor protein p63 regulated 1-like | A0A8I5ZPI8 1xPhospho [T34(100)] | 0.423808 | 2.176021523 |
| P2R1A-PPP2R2A-interacting phosphatase regulator 1 | Q6AYT4 2xPhospho [S142(100);S146(99.5)] | 0.418404 | 2.705741961 |
| Titin;Titin | A0A8I5ZUN3 1xPhospho [S1419(99.4)];A0A8I6A794 1xPhospho [S1374(99.4)] | 0.406625 | 3.113640826 |
| LIM domain 7 | A0A8I6AAI2 1xPhospho [S421(100)] | 0.415037 | 1.72005925 |
| Selenoprotein P | P25236 1xPhospho [S201(100)] | 0.401363 | 1.508486082 |
| Oxysterol-binding protein | A0A8I6AMZ3 1xPhospho [S190(100)] | 0.398549 | 1.80870514 |
| RCSD domain containing 1 | A0A8I5YCC4 1xPhospho [S109(100)] | 0.426625 | 1.38133405 |
| Heterogeneous nuclear ribonucleoprotein K | F8WG62 1xPhospho [S378(100)] | 0.432959 | 1.566816025 |
| Centrosomal protein of 76 kDa | G3V931 1xPhospho [S83(100)] | 0.426815 | 1.528820415 |
| Actin-binding LIM protein 1; Actin-binding LIM protein 1 | A0A8I6A592 3xPhospho [S417(99.1);S420(100);S423(100)];A0A8I6G9J9 3xPhospho [S375(99.1);S378(100);S381(100)] | 0.431157 | 1.321353983 |
| Titin;Titin | A0A8I5ZUN3 1xPhospho [T943(100)];A0A8I6A794 1xPhospho [T898(100)] | 0.437405 | 2.206582374 |
| AHNAK nucleoprotein;AHNAK nucleoprotein | A0A0G2JU96 1xPhospho [S178(99.5)];A0A0G2JUA5 1xPhospho [S178(99.5)] | 0.447459 | 2.931937603 |
| Nuclear pore complex protein Nup93 | Q66HC5 1xPhospho [S52(100)] | 0.440573 | 1.774039745 |
| Supervillin | F1M155 1xPhospho [S486(99.9)] | 0.426815 | 1.948820843 |
| Mediator of RNA polymerase II transcription subunit | D3ZRN2 1xPhospho [S986(100)] | 0.423499 | 1.356437276 |
| Reticulon; Reticulon | A0A8I6GLD1 1xPhospho [S107(100)];Q6IRL3 1xPhospho [S107(100)] | 0.444785 | 1.786651809 |
| Sarcalumenin | A0A8I6G9C9 1xPhospho [Y795(100)] | 0.436099 | 1.720239235 |
| TSC22 domain family, member 3 | A0A8I5ZQF5 2xPhospho [S34(100);S41(98.3)] | 0.437064 | 1.566857506 |
| CDC-like kinase 3 | A0A8I6GM85 1xPhospho [S67(99.5)] | 0.43664 | 1.49494653 |
| Catenin delta 1 | A0A0G2JXM1 1xPhospho [S352(100)] | 0.408806 | 1.308153111 |
| Glucocorticoid receptor | A8IRI3 1xMethylthio [C168];1xPhospho [T170(100)] | 0.453173 | 1.575847511 |
| Eukaryotic translation initiation factor 4 gamma, 3 | A0A0G2JY73 1xPhospho [S314(100)] | 0.463401 | 1.630954151 |
| Forkhead box O4 | A0A096MJF0 1xPhospho [S402(100)] | 0.455195 | 1.35664404 |
| Extended synaptotagmin 2 | D3ZJ32 1xPhospho [S660(98.5)] | 0.450166 | 1.419457274 |
| Myosin light chain kinase, smooth muscle | A0A8I6A5J5 1xPhospho [S1803(100)] | 0.460841 | 2.127109275 |
| A-kinase anchoring protein 2; A-kinase anchoring protein 2 | A0A8I6AAB5 1xPhospho [S318(100)];A0A8I5Y1X4 1xPhospho [S551(100)] | 0.452512 | 1.565710065 |
| Microtubule-associated protein, RP/EB family, member 2 | M0R7M8 1xPhospho [S192(100)] | 0.466781 | 1.817175737 |
| Supervillin | F1M155 1xPhospho [S50(100)] | 0.461331 | 1.591233619 |
| Spliceosome associated factor 1, recruiter of U4/U6.U5 tri-snRNP | A0A8I6ARQ7 1xPhospho [S47(100)] | 0.460841 | 2.161828159 |
| Nebulette; Nebulette | A0A8I6B580 1xPhospho [S810(100)];A0A0G2JWS2 1xPhospho [S903(100)] | 0.454032 | 2.234060259 |
| AHNAK nucleoprotein; AHNAK nucleoprotein | A0A0G2JU96 1xPhospho [S136(100)];A0A0G2JUA5 1xPhospho [S136(100)] | 0.457207 | 1.993596956 |
| Thyroid hormone receptor associated protein 3 | A0A8I5ZUG9 1xPhospho [S640(100)] | 0.444785 | 1.46062443 |
| ITPR interacting domain containing 2 | D3ZLC3 1xPhospho [S90(100)] | 0.445411 | 1.948604723 |
| Reticulon | A0A8I6GLD1 1xPhospho [S295(100)] | 0.461134 | 1.527592313 |
| Cardiomyopathy associated 5 | A0A8I6ALA3 1xPhospho [S242(100)] | 0.445411 | 2.063247253 |
| Beta-glo; Hemoglobin, beta adult major chain; Hemoglobin, beta adult major chain | Q6PDU6 1xPhospho [S126(99.3)];A0A0G2JSW3 1xPhospho [S126(99.3)];A0A8I6AUL4 1xPhospho [S126(99.3)] | 0.467779 | 1.571713862 |
| 5'-nucleotidase, cytosolic IA | D3ZVD3 1xPhospho [S35(100)] | 0.459432 | 2.053827208 |
| Serine/arginine-rich splicing factor 6 | G3V6S8 1xPhospho [S316(99.5)] | 0.47032 | 1.61551939 |
| Inositol-trisphosphate 3-kinase B | P42335 1xPhospho [S247(99.5)] | 0.455679 | 2.608009261 |
| Xin actin-binding repeat-containing protein 2 | Q71LX6 1xPhospho [S2211(100)] | 0.436099 | 2.461137866 |
| PHD finger protein 2 | F1LWX5 1xPhospho [S776(100)] | 0.457473 | 1.497894965 |
| DNA replication licensing factor MCM2 | D3ZP96 1xPhospho [S22(100)] | 0.447459 | 1.317439235 |
| Protein phosphatase 1 regulatory subunit 12A | Q10728 1xPhospho [S507(100)] | 0.459432 | 1.397973566 |
| NADH dehydrogenase [ubiquinone] 1 beta subcomplex subunit 7 | D3ZLT1 1xPhospho [Y62(100)] | 0.482782 | 1.304815226 |
| AHNAK nucleoprotein; AHNAK nucleoprotein | A0A0G2JU96 1xPhospho [T5233(97.9)];A0A0G2JUA5 1xPhospho [T5361(97.9)] | 0.474372 | 1.569252826 |
| Synaptopodin 2 | A0A8I5ZYK1 1xPhospho [S1014(100)] | 0.466568 | 1.302553156 |
| Regulatory associated protein of MTOR, complex 1 | A0A8I6ANV2 1xPhospho [S828(100)] | 0.461331 | 2.265042181 |
| Sorbin and SH3 domain containing 2; Sorbin and SH3 domain containing 2; Sorbin and SH3 domain containing 2 | A0A8I6AA99 1xPhospho [S864(99.4)];A0A8I5ZQ23 1xPhospho [S569(99.4)];A0A8I6ABV1 1xPhospho [S603(99.4)] | 0.485427 | 1.529499606 |
| Calpastatin | P27321 1xPhospho [T173(100)] | 0.453366 | 2.604465217 |
| Cardiomyopathy associated 5 | A0A8I6ALA3 1xPhospho [S698(100)] | 0.468553 | 2.212879358 |
| Synapse associated protein 1 | A0A8I6AEQ4 1xPhospho [T257(100)] | 0.471306 | 1.576936262 |
| Junctional cadherin 5 associated | F1M6T3 1xPhospho [S200(100)] | 0.485427 | 2.994956455 |
| Muscular LMNA-interacting protein | A0A096MJS3 1xPhospho [S260(100)] | 0.477322 | 1.315786361 |
| T-box transcription factor 5 | G3V657 1xPhospho [S41(99.1)] | 0.463401 | 2.180732036 |
| Tensin 1 | A0A8I6AQ89 3xPhospho [S1061(100);S1066(98.8);S1069(100)] | 0.478047 | 1.340892521 |
| Adducin 1; Adducin 1 | A0A8I5Y1E2 D3ZZ99 1xMethylthio [C430] | 0.469485 | 1.418493299 |
| HECT domain E3 ubiquitin protein ligase 4 | A0A8I5ZZM0 1xPhospho [S1581(100)] | 0.47063 | 1.694495409 |
| Multiple PDZ domain crumbs cell polarity complex | A0A8I6G7P8 1xPhospho [S907(100)] | 0.477322 | 1.941479119 |
| Cyclin Y-like 1; Cyclin Y | F1M4U0 1xPhospho [S98(99.6)];A0A8I5ZU57 1xPhospho [S73(99.6)] | 0.466781 | 1.5208322 |
| BCL2 interacting protein 3 | A0A8I6AQ99 1xPhospho [S48(97.9)] | 0.463947 | 1.409411815 |
| Inositol-trisphosphate 3-kinase B | P42335 1xPhospho [S24(100)] | 0.503766 | 2.409743506 |
| Microtubule-associated protein | A0A8I5ZSY4 1xPhospho [S1201(100)] | 0.5025 | 1.836980899 |
| Neurabin-1 | O35867 1xPhospho [S199(100)] | 0.492598 | 3.902094075 |
| mRNA decay activator protein ZFP36 | G3V8K6 1xPhospho [S185(100)] | 0.50696 | 1.580271335 |
| Phosphodiesterase | A0A8I6ASF6 1xPhospho [S204(100)] | 0.49304 | 1.356119897 |
| BCL10, immune signaling adaptor | A0A8I6A3W9 1xPhospho [S119(100)] | 0.496676 | 1.377181023 |
| Transmembrane protein 245 | D3ZR79 2xPhospho [S324(98.6);S/T] | 0.5025 | 2.373403561 |
| FRY like transcription coactivator | A0A8I6A585 1xPhospho [S2286(100)] | 0.499102 | 1.951052411 |
| P2R1A-PPP2R2A-interacting phosphatase regulator 1 | Q6AYT4 1xPhospho [S146(99.6)] | 0.482942 | 1.418173747 |
| EH domain binding protein 1 | A0A8I5ZZB4 1xPhospho [S920(100)] | 0.485427 | 1.373204843 |
| Non-specific serine/threonine protein kinase | A0A0G2KAS8 1xPhospho [S1717(100)] | 0.474909 | 1.580431311 |
| Adenylyl cyclase-associated protein | A0A8I6AQS4 1xPhospho [S189(100)] | 0.510962 | 1.362139625 |
| WW domain containing transcription regulator 1 | Q4V7E6 1xPhospho [S87(99.3)] | 0.519374 | 2.380292168 |
| Dedicator of cytokinesis 2 | A0A8I5ZRT6 1xPhospho [S1684(100)] | 0.514573 | 1.676321419 |
| Erythrocyte membrane protein band 4.1-like 3 | A0A8I6AAL7 1xPhospho [S920(100)] | 0.526748 | 1.455935565 |
| PTPRF interacting protein alpha 1 | D3ZZ81 1xPhospho [T760(99.5)] | 0.526546 | 1.454678314 |
| RCSD domain containing 1 | A0A8I5YCC4 1xPhospho [S242(100)] | 0.498806 | 2.260362687 |
| Nuclear receptor binding protein 2 | A0A0G2JT23 1xPhospho [S207(100)] | 0.510195 | 2.094843632 |
| PNN interacting serine and arginine rich protein | F1MAQ8 1xPhospho [S211(100)] | 0.522841 | 2.195077683 |
| Tankyrase 1 binding protein 1 | D3ZF26 1xPhospho [S1125(100)] | 0.501194 | 2.794322248 |
| G protein pathway suppressor 1 | A0A0G2JT06 1xPhospho [S509(100)] | 0.536599 | 2.827337283 |
| AHNAK nucleoprotein; AHNAK nucleoprotein | A0A0G2JU96 2xPhospho [S213(100);S217(100)];A0A0G2JUA5 2xPhospho [S213(100);S217(100)] | 0.537266 | 2.089167608 |
| Eukaryotic translation elongation factor 1 delta | A0A8I5ZUU1 1xPhospho [S47(100)] | 0.536053 | 2.175134982 |
| Oxidation resistance protein 1 | Q4V8B0 1xPhospho [S194(99.6)] | 0.537028 | 2.486059462 |
| Treacle ribosome biogenesis factor 1 | D4A206 1xPhospho [T946(99.2)] | 0.552541 | 1.651422025 |
| Snta1 protein | B5DFL0 2xPhospho [S194(100);S195(100)] | 0.532014 | 1.965905096 |
| Vacuolar protein sorting 13 homolog C | A0A8I5ZMR7 1xPhospho [S713(99.5)] | 0.537965 | 2.988264643 |
| Caspase-activated deoxyribonuclease inhibitor | Q9JLT3 1xPhospho [S28(100)] | 0.549687 | 1.904920442 |
| Mitotic deacetylase associated SANT domain protein | D4ACA6 1xPhospho [S456(100)] | 0.545161 | 1.800128111 |
| Tripartite motif-containing 54 | A0A8I5ZS50 1xPhospho [S222(100)] | 0.534336 | 1.807335971 |
| Band 4.1-like protein 1 | Q9WTP0 1xPhospho [T475(100)] | 0.525461 | 1.348288845 |
| Non-specific serine/threonine protein kinase | A0A8I5ZMP0 1xPhospho [S75(100)] | 0.551015 | 1.440062473 |
| Palmitoyltransferase ZDHHC5 | Q2THW7 1xPhospho [S554(100)] | 0.535596 | 2.801055946 |
| Protein phosphatase 1 regulatory subunit | A0A0G2K4R1 1xPhospho [S508(100)] | 0.55141 | 2.895998866 |
| Alpha-2-HS-glycoprotein | F1LM19 1xPhospho [S313(100)] | 0.562936 | 2.181496628 |
| Casein kinase 1, alpha 1 | A0A8I6GCP2 1xPhospho [T349(100)] | 0.5603 | 2.227158771 |
| Cordon-bleu WH2 repeat protein-like 1 | F1M124 1xPhospho [S1159(100)] | 0.551796 | 1.577782775 |
| AHNAK nucleoprotein;AHNAK nucleoprotein | A0A0G2JU96 3xPhospho [S211(100);S217(100);S222(100)];A0A0G2JUA5 3xPhospho [S211(100);S217(100);S222(100)] | 0.552541 | 1.477032968 |
| Transcription factor Jun | P17325 1xPhospho [S63(100)] | 0.542527 | 2.075616529 |
| Ankyrin 1 | A0A8I6GAS7 1xPhospho [S1490(100)] | 0.555215 | 1.934259088 |
| orbin and SH3 domain containing 2; Sorbin and SH3 domain containing 2; Sorbin and SH3 domain containing 2 | A0A8I6AA99 1xPhospho [S433(100)];A0A8I5ZQ23 1xPhospho [S185(100)];A0A8I6ABV1 1xPhospho [S200(100)] | 0.553598 | 2.691049102 |
| Serine/threonine-protein phosphatase 1 regulatory subunit 10 | A0A0G2K3G9 1xPhospho [S313(100)] | 0.530515 | 1.776810435 |
| Sorbin and SH3 domain containing 2; Sorbin and SH3 domain containing 2; Sorbin and SH3 domain containing 2 | A0A8I6AA99 1xPhospho [S524(100)];A0A8I5ZQ23 1xPhospho [S276(100)];A0A8I6ABV1 1xPhospho [S291(100)] | 0.555519 | 1.941765504 |
| AHNAK nucleoprotein; AHNAK nucleoprotein | A0A0G2JU96 1xPhospho [S94(100)];A0A0G2JUA5 1xPhospho [S94(100)] | 0.547488 | 1.845673102 |
| Avian erythroblastosis virus E-26 (V-ets) oncogene | Q6IMZ7 1xPhospho [S215(100)] | 0.555519 | 2.265825692 |
| Growth factor inhibitor | B1A2U8 1xPhospho [S1185(100)] | 0.564498 | 2.303948186 |
| FYN binding protein 1 | A0A8I6G9A9 1xPhospho [S557(100)] | 0.543142 | 2.426928129 |
| Pre-mRNA-splicing regulator WTAP | D3ZPY0 1xPhospho [T298(98.7)] | 0.561116 | 1.480221809 |
| Cardiomyopathy associated 5 | A0A8I6ALA3 1xPhospho [T2851(99.2)] | 0.567041 | 2.037040039 |
| SH2 domain containing 3C | A0A8I5ZME7 1xPhospho [S282(100)] | 0.577976 | 1.885633118 |
| RCSD domain containing 1 | A0A8I5YCC4 1xPhospho [S149(98)] | 0.57836 | 1.843568607 |
| Reticulon | A0A8I6GLD1 2xPhospho [S425(100);T429(99)] | 0.584963 | 1.494480731 |
| Anaphase-promoting complex subunit 2 | F7EWF8 1xPhospho [S548(100)] | 0.584963 | 1.613185806 |
| Proline-rich basic protein 1 | F1M6Z4 1xPhospho [S413(100)] | 0.596367 | 1.564487909 |
| Proline-rich basic protein 1 | F1M6Z4 1xPhospho [S777(100)] | 0.557482 | 2.042914526 |
| Matrin 3 | A0A0G2JSR7 1xPhospho [S188(100)] | 0.57289 | 1.50244508 |
| Sarcoplasmic/endoplasmic reticulum calcium;Ca2+/Mg2+ ATPase | P11507 1xPhospho [S504(100)];Q63080 1xMethylthio [C11];1xPhospho [S17(100)] | 0.569856 | 1.788615921 |
| H1.2 linker histone, cluster member; H1.3 linker histone, cluster member; Histone H1.4 | A0A0G2K654 1xPhospho [S36(100)];M0R7B4 1xPhospho [S37(100)];P15865 1xPhospho [S36(100)] | 0.565597 | 1.467341324 |
| Cardiomyopathy associated 5 | A0A8I6ALA3 1xPhospho [S242(100)] | 0.584963 | 3.267936607 |
| G-protein signaling modulator 1 | A0A8I5ZZF6 1xPhospho [S673(100)] | 0.574694 | 1.662133394 |
| TSC22 domain family, member 3 | A0A8I5ZQF5 1xPhospho [S41(100)] | 0.599038 | 1.32655912 |
| Muscular LMNA-interacting protein | A0A096MJS3 1xPhospho [S571(99.1)] | 0.597241 | 2.451105747 |
| Dynamin-1-like protein | O35303 1xPhospho [S635(100)] | 0.584963 | 1.325749985 |
| Dynein light intermediate chain | G3V7G0 2xPhospho [S510(100);T512(98.7)] | 0.59368 | 1.632022719 |
| Synaptopodin;Synaptopodin | A0A8I6AKF5 1xPhospho [S454(100)];A0A0H2UHQ9 1xPhospho [S569(100)] | 0.621488 | 3.269746506 |
| Treacle ribosome biogenesis factor 1 | D4A206 1xPhospho [S1228(100)] | 0.576192 | 3.104807859 |
| Plakophilin-2 | A0A8I6A597 1xPhospho [S564(100)] | 0.609416 | 1.872015534 |
| Nascent polypeptide associated complex subunit alpha | M0R9L0 1xPhospho [S1354(97.8)] | 0.602665 | 1.562779063 |
| Cardiomyopathy associated 5 | A0A8I6ALA3 1xPhospho [S242(100)] | 0.596103 | 1.353448186 |
| Tyrosine-protein phosphatase non-receptor type | A0A8I5ZUL2 1xPhospho [S461(100)] | 0.612977 | 1.572976808 |
| TPD52 like 1 | Q499Q2 1xPhospho [S137(100)] | 0.59048 | 2.785642027 |
| Titin | A0A8I5ZUN3 1xPhospho [S34581(100)] | 0.592825 | 3.191941135 |
| Talin 2 | D4A3B0 1xPhospho [S1907(99.2)] | 0.584963 | 2.213714869 |
| Peroxisomal ATPase PEX1 | D3ZZB2 1xPhospho [S1181(100)] | 0.620586 | 2.587918026 |
| Bridging integrator 2 | A0A8I5ZY98 1xPhospho [S420(100)] | 0.63743 | 2.161905764 |
| Metadherin | A0A8I5Y7K9 1xPhospho [S424(100)] | 0.629357 | 1.328822391 |
| Sodium/hydrogen exchanger 1 | P26431 1xPhospho [S801(100)] | 0.612977 | 1.43701878 |
| Nipped-B protein | A0A0G2K0J4 1xPhospho [S872(100)] | 0.625604 | 1.342691812 |
| Protein NDRG2 | A0A0G2K3T7 1xPhospho [S358(99.2)] | 0.633218 | 1.5109869 |
| Myosin IXb | A0A0G2JZQ4 1xPhospho [T1311(100)] | 0.628031 | 1.360282512 |
| Tensin 1 | A0A8I6AQ89 1xPhospho [S929(100)] | 0.63941 | 1.772974878 |
| AHNAK nucleoprotein; AHNAK nucleoprotein | A0A0G2JU96 1xPhospho [S739(100)];A0A0G2JUA5 1xPhospho [S889(100)] | 0.657112 | 1.379856826 |
| Trafficking kinesin protein 1 | A0A8I6AI05 1xPhospho [S228(99.6)] | 0.625604 | 1.42850403 |
| RPTOR independent companion of MTOR, complex 2 | A0A8I5ZNG4 1xPhospho [S1486(99.6)] | 0.640104 | 2.052914617 |
| Afadin, adherens junction formation factor | A0A8I5ZLU6 1xPhospho [S1705(100)] | 0.647698 | 1.856390637 |
| Junctional cadherin 5 associated | F1M6T3 1xPhospho [S1281(100)] | 0.649503 | 2.052613486 |
| Gap junction protein | A0A654ICE6 1xPhospho [S282(96.5)] | 0.650058 | 1.30414172 |
| Tnnt2 protein | B1WBR4 1xPhospho [S191(100)] | 0.671377 | 2.107527604 |
| Band 4.1-like protein 1 | Q9WTP0 1xPhospho [T475(100)] | 0.625152 | 1.675834288 |
| CREB regulated transcription coactivator 3 | F1LVL6 1xPhospho [S316(100)] | 0.633461 | 1.648193568 |
| Microtubule-associated protein 1B | A0A8I5ZWW4 1xPhospho [S339(99.5)] | 0.658963 | 3.603870339 |
| BCL2-associated transcription factor 1, isoform | B1WC16 1xPhospho [S690(100)] | 0.627088 | 3.021266439 |
| Muscular LMNA-interacting protein | A0A096MJS3 1xPhospho [T148(100)] | 0.63743 | 2.49258484 |
| Golgi brefeldin A resistant guanine nucleotide | F1M8X9 1xPhospho [S1783(99.6)] | 0.68054 | 2.480276783 |
| RGD1308612 protein | Q4KLK2 1xPhospho [S66(100)] | 0.674014 | 1.910264964 |
| Xin actin-binding repeat-containing protein 2 | Q71LX6 1xPhospho [S1573(100)] | 0.670432 | 2.521290171 |
| Nucleoporin 188 | F1LRC6 1xPhospho [S1714(99.3)] | 0.678072 | 1.594056633 |
| Dystrophin | A0A8I6GKZ8 1xPhospho [S3528(97.8)] | 0.663818 | 1.678555477 |
| F-actin monooxygenase | A0A8I6A4K7 1xPhospho [S1420(100)] | 0.659925 | 2.152620799 |
| Transformation/transcription domain-associated protein | A0A8I6GLC1 1xPhospho [S2032(100)] | 0.665264 | 1.612866324 |
| Non-specific serine/threonine protein kinase | A0A0G2JUP3 1xPhospho [S6384(100)] | 0.671377 | 1.571429366 |
| Cardiomyopathy associated 5 | A0A8I6ALA3 1xPhospho [S242(100)] | 0.697437 | 1.727697838 |
| Sodium/hydrogen exchanger 1 | P26431 2xPhospho [S790(100);S/T] | 0.663951 | 2.162490065 |
| Lamin A/C | G3V8L3 2xPhospho [S22(98.7);S/T] | 0.669851 | 1.397510556 |
| Ragulator complex protein LAMTOR1 | A0A8I5Y5M8 1xPhospho [T28(100)] | 0.652077 | 1.801355845 |
| Transcription elongation factor A protein 1 | Q4KLL0 1xPhospho [S135(98.3)] | 0.671377 | 2.600581985 |
| Phosphoinositide phospholipase C | A0A8I6AGE0 1xPhospho [S512(100)] | 0.652077 | 1.33232275 |
| DNA-directed RNA polymerase III subunit RPC3 | Q5XIL3 2xPhospho [S204(100);S205(100)] | 0.649814 | 1.868129691 |
| Microtubule-associated protein 1A | G3V7U2 1xPhospho [S1643(100)] | 0.683526 | 1.976088236 |
| Dystonin | A0A8I5ZQD4 1xPhospho [S2681(100)] | 0.646363 | 1.628868218 |
| Ubiquitin-conjugating enzyme E2O | A0A8I6A0W6 1xPhospho [S839(100)] | 0.694374 | 2.323964162 |
| Tripartite motif-containing 54 | A0A8I5ZS50 1xPhospho [S222(100)] | 0.669851 | 2.422429408 |
| G-rich RNA sequence binding factor 1 | F1LRK4 1xPhospho [S226(100)] | 0.695145 | 1.974682577 |
| Splicing factor 3b, subunit 2 | D3ZMS1 2xPhospho [S326(100);S] | 0.706269 | 2.75131243 |
| Dolichyl-diphosphooligosaccharide--protein | A0A8I5ZNT8 1xPhospho [S444(99.5)] | 0.702614 | 1.576366681 |
| KN motif and ankyrin repeat domains 1 | A0A8I6GML0 1xPhospho [T1371(99.4)] | 0.683143 | 2.654921259 |
| Chromobox 3 | Q5RJK5 1xPhospho [S93(100)] | 0.705553 | 2.43457477 |
| Palladin-like 1 | A0A8I6ADH4 1xPhospho [S487(100)] | 0.714598 | 1.734090214 |
| Microtubule associated scaffold protein 2 | A0A8J8YSZ5 1xPhospho [S258(100)] | 0.713119 | 1.522818435 |
| [histone H3]-lysine(4) N-methyltransferase | A0A0G2JVD6 1xPhospho [S4745(100)] | 0.732716 | 1.675528923 |
| Ankyrin 2 | F1M9N9 1xPhospho [T3736(99.4)] | 0.726982 | 2.304734919 |
| RAB11 family interacting protein 5 | A0A0G2K1W1 1xPhospho [S631(100)] | 0.736966 | 1.644605963 |
| Rho GTPase activating protein 1 | A0A0G2JYR5 1xPhospho [S124(99.6)] | 0.729352 | 1.939959696 |
| Nascent polypeptide associated complex | M0R9L0 1xPhospho [S770(100)] | 0.736966 | 2.20471208 |
| Protocadherin beta 22 | G3V8N1 1xPhospho [T79(100)] | 0.73252 | 2.898109565 |
| Caldesmon 1 | A0A8I6GCL3 1xPhospho [S746(99.5)] | 0.745427 | 1.627204274 |
| Mitochondrial antiviral-signaling protein | Q66HG9 1xPhospho [S458(100)] | 0.765535 | 1.652871877 |
| 5'-AMP-activated protein kinase subunit beta-1 | P80386 1xPhospho [S108(100)] | 0.757143 | 1.538471578 |
| Muscular LMNA-interacting protein | A0A096MJS3 1xPhospho [S686(100)] | 0.766801 | 1.390221754 |
| A-kinase anchor protein SPHKAP | P0C6C0 2xPhospho [T1360(98.9);S1369(100)] | 0.751321 | 1.58912429 |
| AT hook containing transcription factor 1 | A0A8I5ZM44 1xPhospho [S1898(99.6)] | 0.77596 | 1.510466743 |
| LIM domain binding 3;LIM domain binding 3;LIM domain binding 3 | A0A0G2JXR0 1xPhospho [S254(100)];A0A8I5Y5H4 1xPhospho [S299(100)];A0A0G2K2C4 1xPhospho [S207(100)] | 0.767827 | 1.731824143 |
| Splicing factor SWAP | A0A0G2JZ87 1xPhospho [S954(100)] | 0.774119 | 1.768524549 |
| Pleckstrin homology-like domain, family B, member 1; Pleckstrin homology-like domain, family B, member 1 | A0A8I6A9F1 1xPhospho [S490(100)];A0A0G2JV32 1xPhospho [S490(100)] | 0.77961 | 3.107440348 |
| Spectrin beta chain | Q6XD99 1xPhospho [S2155(100)] | 0.76833 | 1.680894952 |
| Heat shock 70 kDa protein 4 | F1LRV4 1xPhospho [S47(100)] | 0.75269 | 2.071978141 |
| Inositol 1,4,5-trisphosphate receptor | F1LQR8 1xPhospho [S1160(100)] | 0.766801 | 1.567440199 |
| Glycogen [starch] synthase | A0A8I5ZWA2 1xPhospho [S589(100)] | 0.776225 | 1.39155903 |
| Tubulin-glutamate carboxypeptidase | G3V8G1 1xPhospho [S1161(99)] | 0.803308 | 1.348513126 |
| Testis expressed 2 | G3V9L1 2xPhospho [T269(99.3);S270(100)] | 0.802061 | 1.853737798 |
| Plectin 11 | Q6S395 1xPhospho [S1427(99.7)] | 0.77444 | 1.480364933 |
| TSC22 domain family, member 3 | A0A8I5ZQF5 1xPhospho [S72(100)] | 0.782409 | 1.722837645 |
| Actin-binding LIM protein 1 | A0A8I6A592 1xPhospho [S293(99.7)] | 0.777608 | 1.677148003 |
| Zyxin | A0A8I6GKY5 1xPhospho [S374(100)] | 0.78958 | 1.69766616 |
| Eukaryotic translation initiation factor 3 subunit B | Q4G061 1xPhospho [S105(100)] | 0.777608 | 4.491168345 |
| RNA binding motif protein 33 | A0A8I6AN05 1xPhospho [S774(100)] | 0.794251 | 1.947837444 |
| Filamin-C | D3ZHA0 1xPhospho [S2625(100)] | 0.780219 | 2.423470624 |
| Nuclear cap-binding protein subunit 1 | Q56A27 1xPhospho [S7(100)] | 0.79518 | 1.42289546 |
| Integrator complex subunit 6 | A0A8I6G5G2 1xPhospho [S747(100)] | 0.818553 | 1.651209616 |
| SMCR8-C9orf72 complex subunit | D3ZJR6 1xPhospho [S416(100)] | 0.825971 | 4.596923923 |
| Baculoviral IAP repeat-containing 6 | F1LY70 1xPhospho [S235(99.2)] | 0.800691 | 1.83841784 |
| Thyroid hormone receptor associated protein 3 | A0A8I5ZUG9 2xPhospho [S238(100);S243(100)] | 0.810966 | 3.396580862 |
| Myopalladin | D4A7X7 1xPhospho [S660(99.2)] | 0.83399 | 1.305275093 |
| E3 ubiquitin-protein ligase RNF34 | Q6AYH3 1xPhospho [S263(100)] | 0.818162 | 1.695399265 |
| Branched-chain-amino-acid aminotransferase, mitochondrial | O35854 1xPhospho [S385(99.5)] | 0.817797 | 2.601102462 |
| Cardiomyopathy associated 5 | A0A8I6ALA3 1xPhospho [S2973(100)] | 0.834414 | 3.680095163 |
| Nascent polypeptide associated complex subunit alpha | M0R9L0 1xPhospho [S1845(100)] | 0.856636 | 1.650809441 |
| Synaptosome associated protein 91 | A0A8I5ZLY5 1xPhospho [S603(100)] | 0.827163 | 2.237729571 |
| Myeloid leukemia factor 1 | A0A8I6AES2 1xPhospho [S34(100)] | 0.839535 | 2.968072796 |
| Protein NDRG2 | A0A0G2K3T7 2xPhospho [S352(100);S358(99)] | 0.845025 | 2.894219678 |
| Aminoacyl tRNA synthase complex-interacting multifunctional protein 2 | Q32PX2 1xPhospho [S36(99.3)] | 0.871676 | 4.624595905 |
| LIM and calponin homology domains 1 | F1M392 1xPhospho [S303(99.3)] | 0.861294 | 1.55497844 |
| Splicing factor 3b, subunit 2 | D3ZMS1 1xPhospho [S326(100)] | 0.874469 | 1.631812901 |
| Ubiquitin carboxyl-terminal hydrolase CYLD | A0A8I6AAK9 1xPhospho [S345(100)] | 0.847997 | 1.445833834 |
| Chromobox 3 | Q5RJK5 1xPhospho [S93(100)] | 0.852774 | 1.797401027 |
| PPARGC1 and ESRR induced regulator, muscle 1 | D3ZH76 1xPhospho [S403(100)] | 0.871267 | 1.723827299 |
| Transgelin-2 | Q5XFX0 1xPhospho [S163(100)] | 0.882164 | 1.948771675 |
| Zyxin | A0A8I6GKY5 1xPhospho [S438(100)] | 0.870717 | 1.503588814 |
| AT hook containing transcription factor 1 | A0A8I5ZM44 1xPhospho [S1309(100)] | 0.882164 | 1.822087036 |
| KN motif and ankyrin repeat domain-containing protein 2 | D3ZD05 1xPhospho [S561(100)] | 0.899473 | 4.618123175 |
| EH domain binding protein 1-like 1 | A0A0G2K6R8 1xPhospho [S1363(100)] | 0.872995 | 1.939553236 |
| Nuclear receptor coactivator 5 | A0A8I6AHM9 1xPhospho [S60(100)] | 0.889297 | 1.558231356 |
| Inositol-trisphosphate 3-kinase B | P42335 1xPhospho [S42(100)] | 0.908852 | 1.379367192 |
| Dynamin-1-like protein | O35303 1xPhospho [S635(100)] | 0.921997 | 2.221714839 |
| Coronin-7 | O35828 1xPhospho [S459(100)] | 0.91427 | 3.089043932 |
| Formin homology 2 domain containing 3 | F1LRX2 1xPhospho [S510(100)] | 0.893085 | 1.703418178 |
| Cleavage and polyadenylation specificity factor subunit 7 | Q5XI29 2xPhospho [S185(99.4);S188(100)] | 0.924712 | 1.583359975 |
| Ankyrin 1 | A0A8I6GAS7 1xPhospho [S1827(99.6)] | 0.966317 | 2.111247684 |
| 60 kDa heat shock protein, mitochondrial | P63039 1xPhospho [S232(99.4)] | 0.985786 | 1.532688945 |
| Pinin | A0A8I6AKS0 1xPhospho [S66(100)] | 0.975752 | 1.947307881 |
| FERM domain containing 5 | F1LT14 1xPhospho [S396(99.5)] | 0.988685 | 1.814166375 |
| Titin;Cardiac titin N2B isoform | A0A8I5ZUN3 1xPhospho [S4111(100)];Q7TMZ9 1xPhospho [S595(100)] | 1 | 2.009388771 |
| Cleavage and polyadenylation specificity factor subunit 7 | Q5XI29 1xPhospho [S185(99.4)] | 1 | 2.004754089 |
| Sperm antigen with calponin homology and coiled-coil domains 1 | A0A0G2K5D7 1xPhospho [S424(97.9)] | 1.0199 | 1.353795054 |
| Smoothelin;Smoothelin | A0A8I5ZNI1 1xPhospho [S641(99.6)];A0A8I6A098 1xPhospho [S318(99.6)] | 1.010569 | 3.160995431 |
| AHNAK nucleoprotein; AHNAK nucleoprotein | A0A0G2JU96 1xPhospho [T4835(100)];A0A0G2JUA5 1xPhospho [T4963(100)] | 1.062122 | 1.455962567 |
| CDKN2A-interacting protein | Q5U2X0 1xPhospho [S124(99.7)] | 1.064955 | 1.512691942 |
| LIM domain and actin binding 1 | A0A8I6AM55 1xPhospho [S715(100)] | 1.074001 | 1.336705583 |
| Microtubule-associated protein, RP/EB family, member 2 | M0R7M8 1xPhospho [S221(100)] | 1.031027 | 3.820251928 |
| Torsin 1A interacting protein 2 | A0A8I6AJQ9 1xPhospho [S88(100)] | 1.067745 | 1.326124736 |
| Protein phosphatase 1, regulatory subunit 3A | D3ZM60 1xPhospho [S600(100)] | 1.04891 | 1.883052839 |
| Pleckstrin homology domain containing A6 | A0A8I6AI30 1xPhospho [S833(99.3)] | 1.071791 | 1.344246453 |
| Cardiac-enriched FHL2-interacting protein | M0RD54 3xPhospho [S676(100);S678(100);S/T] | 1.08092 | 1.407620131 |
| Torsin-1A-interacting protein 1 | Q5PQX1 1xPhospho [S242(100)] | 1.066089 | 1.486324355 |
| Splicing factor 3b, subunit 2 | D3ZMS1 1xPhospho [S326(100)] | 1.097297 | 1.855096246 |
| Stathmin | A0A8I5ZX99 1xPhospho [S37(100)] | 1.079727 | 1.924216984 |
| PPARGC1 and ESRR induced regulator, muscle 1 | D3ZH76 1xPhospho [S257(100)] | 1.097847 | 1.666361298 |
| UBX domain protein 4 | A0A8I5ZL25 1xPhospho [T156(99.3)] | 1.124696 | 1.736554973 |
| Huntingtin | A0A8I6AXC3 1xPhospho [S398(100)] | 1.119299 | 1.884728799 |
| Titin;Titin | A0A8I5ZUN3 1xPhospho [S3374(100)];A0A8I6A794 1xMethylthio [C3326];1xPhospho [S3329(100)] | 1.120294 | 3.232477718 |
| PPARGC1 and ESRR induced regulator, muscle 1 | D3ZH76 1xPhospho [S257(100)] | 1.117184 | 2.722691365 |
| ranscription intermediary factor 1-beta | O08629 1xPhospho [S474(100)] | 1.129283 | 1.77154316 |
| AHNAK nucleoprotein;HNAK nucleoprotein | A0A0G2JU96 1xPhospho [S3880(100)];A0A0G2JUA5 1xPhospho [S4002(100)] | 1.156504 | 1.916479583 |
| Myomesin 1 | A0A8I6GBP8 1xPhospho [S390(100)] | 1.159657 | 1.573212985 |
| Transforming, acidic coiled-coil containing protein 2 | A0A0G2K598 1xMethylthio [C939] | 1.129283 | 1.711290485 |
| Desmin | P48675 1xPhospho [S45(100)] | 1.156119 | 2.333012915 |
| Cardiomyopathy associated 5 | A0A8I6ALA3 1xPhospho [S1808(99.2)] | 1.195551 | 1.543532548 |
| Monoglyceride lipase | A0A8I5ZPF3 1xPhospho [S217(100)] | 1.186878 | 2.019289101 |
| Transcription intermediary factor 1-beta | O08629 1xPhospho [S474(100)] | 1.190103 | 2.274505975 |
| Transcriptional coactivator YAP1 | Q2EJA0 1xPhospho [S112(99.2)] | 1.196397 | 1.949490407 |
| Zinc finger CCCH domain-containing protein 14 | D4A652 1xPhospho [S309(100)] | 1.210567 | 2.408817175 |
| Insulin receptor substrate 2 | F1MAL5 1xPhospho [S617(99.4)] | 1.216811 | 1.526566211 |
| Xin actin-binding repeat-containing protein 2 | Q71LX6 1xPhospho [S2953(99.4)] | 1.233797 | 2.661877543 |
| Syntaxin-8 | Q9Z2Q7 1xPhospho [S102(100)] | 1.280108 | 1.42722208 |
| Phospholipase A2, activating protein | A0A8I6A2M3 1xPhospho [S318(100)] | 1.286304 | 2.070208842 |
| Alpha-crystallin B chain | P23928 1xPhospho [S59(100)] | 1.309328 | 2.126868665 |
| Choline-phosphate cytidylyltransferase A | P19836 3xPhospho [S315(100);S319(100);S/T] | 1.308122 | 1.632616174 |
| Dystrophin | A0A8I6GKZ8 2xPhospho [S3602(100);S3603(100)] | 1.403999 | 4.994011531 |
| Tripartite motif-containing protein 55 | Q5PQN5 1xPhospho [S170(100)] | 1.559427 | 1.76042781 |
| High-mobility group nucleosome binding domain 1 | Q5U1W8 1xPhospho [S7(100)] | 1.627088 | 1.92380254 |
| Phosphatidylinositol-4-phosphate 3-kinase, catalytic subunit type 2 alpha | D3ZTF6 1xPhospho [S261(100)] | 1.736966 | 2.420955923 |
| Mitochondrial import receptor subunit TOM20 homolog | Q62760 2xPhospho [S135(100);S138(100)] | 1.951978 | 4.416266471 |
