# Supplementary material for: Ischemic preconditioning affects phosphosites and accentuates myocardial stunning while reducing infarction size in rats
Source: Front Cardiovasc Med. 2024 Mar 15;11:1376367. doi: 10.3389/fcvm.2024.1376367 (PMC10978780; doi:10.3389/fcvm.2024.1376367)
Supplement: Supplementary file 2 [file Table2.docx]

**Supplementary Table 2:** Downregulated phosphopeptides in the preconditioned group relative to the non-preconditioned group.

| **Protein Names** | **Modifications in Master Proteins** | **Log2FC** | **-Log Welch's T-test p-value** |
| --- | --- | --- | --- |
| Heat shock protein HSP 90-beta | P34058 1xPhospho [S226(100)] | -1.8574 | 1.53673509 |
| Eukaryotic translation initiation factor 3 subunit C | B5DFC8 2xPhospho [S9(98.2);S/T/Y] | -1.30256 | 1.364509954 |
| Nuclear casein kinase and cyclin-dependent kinase substrate 1 | A0A0G2K7X3 1xPhospho [S273(100)] | -1.2578 | 1.932688955 |
| Ankyrin 2 | F1M9N9 1xPhospho [S825(100)] | -0.98682 | 2.39856751 |
| calcium/calmodulin-dependent protein kinase | A0A8I6AD45 1xPhospho [T287(100)] | -0.97763 | 1.421368669 |
| Connector enhancer of kinase suppressor of Ras 1 | G3V8W8 2xPhospho [S535(100);T537(100)] | -0.94753 | 1.309734779 |
| AHNAK nucleoprotein; AHNAK nucleoprotein | A0A0G2JU96 1xPhospho [S5262(100)];A0A0G2JUA5 1xPhospho [S5390(100)] | -0.82947 | 2.601387671 |
| Histone H4 | P62804 1xPhospho [S48(100)] | -0.79141 | 1.680363788 |
| Glycogen [starch] synthase | A0A8I5ZWA2 5xPhospho [S577(100);S581(99.7);S588(93.1);S589(99.6);S593(100)] | -0.79077 | 1.869014051 |
| Lysosomal cobalamin transport escort protein LMBD1 | Q6AZ61 2xPhospho [S525(100);S528(100)] | -0.76471 | 2.469639045 |
| WD repeat domain 20 | A0A8I6G8P3 1xPhospho [S286(100)] | -0.75384 | 1.766082589 |
| Ankyrin 2 | F1M9N9 1xPhospho [S825(100)] | -0.76356 | 1.912798246 |
| Eva-1 homolog B | B2RZB3 2xPhospho [S133(98.4);T134(100)] | -0.74296 | 1.668946402 |
| RNA polymerase-associated protein LEO1 | Q641X2 1xPhospho [S291(100)] | -0.72348 | 2.008634911 |
| Heat shock protein HSP 90-alpha | P82995 1xPhospho [S263(100)] | -0.71272 | 1.814314149 |
| Phosphoinositide phospholipase C | A0A8I6AGE0 1xPhospho [S451(100)] | -0.71242 | 2.301302443 |
| Heat shock protein HSP 90-alpha | P82995 1xPhospho [S263(100)] | -0.7137 | 2.696127045 |
| Elongation factor 1-beta | B5DEN5 1xPhospho [S106(100)] | -0.70192 | 2.173888492 |
| SH3 and PX domains 2A | A0A8I6AKV6 1xPhospho [S708(100)] | -0.70134 | 2.900140655 |
| Crumbs cell polarity complex component | A0A8I5ZJD2 1xPhospho [S647(100)] | -0.69282 | 1.592177191 |
| Non-specific serine/threonine protein kinase | A0A0G2JUP3 1xPhospho [T7153(99)] | -0.69632 | 2.772910381 |
| Dual specificity phosphatase 27 | D3ZRM0 1xPhospho [S297(99.2)] | -0.71187 | 1.586762819 |
| Supervillin | F1M155 2xPhospho [S298(100);S299(100)] | -0.67807 | 1.773920118 |
| General vesicular transport factor p115 | A0A8I6A7H7 1xPhospho [S938(100)] | -0.68012 | 2.958022866 |
| Myomesin 1 | A0A8I6GBP8 1xPhospho [S159(100)] | -0.66838 | 2.467522964 |
| Non-specific serine/threonine protein kinase | A0A8I6AEW1 1xPhospho [S1313(100)] | -0.68203 | 1.889879354 |
| EMAP like 3 | D4A4R1 1xPhospho [S157(99.1)] | -0.66935 | 1.356530605 |
| Caveolae-associated protein 1 | P85125 1xPhospho [S302(100)] | -0.67105 | 1.791740648 |
| Dual specificity phosphatase 27 | D3ZRM0 2xPhospho [S552(100);S559(100)] | -0.66855 | 1.60722361 |
| Charged multivesicular body protein 3 | Q8CGS4 1xPhospho [S200(100)] | -0.65992 | 2.232913833 |
| MARCKS-related protein | Q9EPH2 1xPhospho [S104(100)] | -0.6567 | 1.606304502 |
| Smoothelin | A0A8I6A098 1xPhospho [S134(99.4)] | -0.64669 | 1.415848559 |
| ARF GTPase-activating protein GIT2 | Q66H91 1xPhospho [S586(99.4)] | -0.65016 | 2.208708157 |
| Succinate-CoA ligase subunit beta | B2RZ24 1xPhospho [S151(100)] | -0.64307 | 1.915803746 |
| Cardiac phospholamban | P61016 2xPhospho [S16(100);T17(100)] | -0.63631 | 1.331681236 |
| WASH complex subunit 2C ; WASH complex subunit 2C | F1LPG9 1xPhospho [S71(99)];A0A8I6AM40 1xPhospho [S157(99)] | -0.63113 | 1.668999305 |
| GTP-binding protein 1 | D2XV59 2xPhospho [S44(100);S47(100)] | -0.62532 | 3.236313085 |
| Nuclear casein kinase and cyclin-dependent kinase substrate 1 | A0A0G2K7X3 1xPhospho [S157(100)] | -0.62803 | 1.790797764 |
| LIM domain 7; LIM domain 7 | A0A8I6AAI2 1xPhospho [S886(99.4)];A0A8I5ZR48 1xPhospho [S580(99.4)] | -0.60666 | 1.56026402 |
| Charged multivesicular body protein 3 | Q8CGS4 1xPhospho [S200(100)] | -0.59036 | 1.992927147 |
| Non-specific serine/threonine protein kinase | A0A8I6A6A2 2xPhospho [S645(98.9);S649(100)] | -0.59337 | 2.125751435 |
| RAS protein activator like 2 | A0A8I5ZWM6 1xPhospho [S1011(100)] | -0.59946 | 1.513947706 |
| Dynamin-binding protein | M0R4F8 1xPhospho [S481(99.5)] | -0.59767 | 2.523188929 |
| Actin dependent regulator of chromatin | A0A8I6G5D7 3xPhospho [S1535(100);S1540(100);S1551(100)] | -0.59418 | 1.565889217 |
| Taxilin beta | A0A0G2K2T1 2xPhospho [T481(100);S482(100)] | -0.58496 | 1.921491657 |
| Ankyrin 2 | F1M9N9 1xPhospho [S3804(99.5)] | -0.57886 | 1.776786286 |
| Nuclear casein kinase and cyclin-dependent kinase substrate 1 | A0A0G2K7X3 2xPhospho [S176(100);S188(100)] | -0.56478 | 1.493649415 |
| Glutamine--fructose-6-phosphate aminotransferase [isomerizing] 1 | P82808 1xPhospho [S243(99.6)] | -0.57116 | 2.694577807 |
| D4ADB4 | D4ADB4 1xPhospho [S164(100)] | -0.57424 | 2.098744993 |
| Bcl-2-interacting death suppressor; BAG cochaperone 3 | Q156J1 2xPhospho [T288(100);S294(100)];Q5U2U8 1xMethylthio [C292];2xPhospho [T288(100);S294(100)] | -0.56071 | 1.67977712 |
| Synaptopodin;Synaptopodin | A0A8I6AKF5 2xPhospho [S627(100);T629(99.5)];A0A0H2UHQ9 1xMethylthio [C740];2xPhospho [S742(100);T744(99.5)] | -0.55394 | 2.026686167 |
| PDZ and LIM domain 4 | M0R4H5 2xPhospho [S119(100);S124(100)] | -0.56937 | 1.417493946 |
| Tyrosine-protein phosphatase non-receptor | Q62728 2xPhospho [S710(100);S711(100)] | -0.53766 | 3.352611478 |
| Histone acetyltransferase | A0A0G2K9F0 1xPhospho [S56(100)] | -0.55199 | 1.330610135 |
| Methylosome subunit pICln | Q6P9X1 1xPhospho [S100(100)] | -0.54909 | 1.667331033 |
| LRR binding FLII interacting protein 2 | A0A8I5ZUL9 1xPhospho [S303(100)] | -0.53842 | 1.314949538 |
| Microtubule-associated protein | F1LST4 1xPhospho [S204(100)] | -0.55706 | 1.539026177 |
| Heat shock protein HSP 90-alpha | P82995 1xPhospho [S231(100)] | -0.55639 | 1.425668745 |
| Caveolae-associated protein 2 | Q66H98 1xPhospho [S326(100)] | -0.52356 | 1.977675695 |
| ETS proto-oncogene 1 | A0A8I5ZWK1 1xPhospho [S295(100)] | -0.53703 | 2.118523322 |
| Transcription factor 20 | D3ZG21 1xPhospho [T1703(99.5)] | -0.52693 | 1.423344242 |
| General vesicular transport factor p115 | A0A8I6A7H7 1xPhospho [S938(100)] | -0.54362 | 2.597090736 |
| Non-specific serine/threonine protein kinase | A0A0G2JUP3 2xPhospho [S5468(100);T5472(100)] | -0.53051 | 1.381136072 |
| LPS responsive beige-like anchor protein | A0A0G2JYI0 1xPhospho [S1162(99.3)] | -0.52284 | 3.924826387 |
| Biorientation of chromosomes in cell division 1-like 1 | A0A8I6A9H5 2xPhospho [S480(100);S482(100)] | -0.53703 | 2.89373624 |
| Pre-mRNA-splicing factor 38A | D3ZGL5 2xPhospho [S193(100);S194(100)] | -0.52356 | 2.894294299 |
| Nascent polypeptide associated complex subunit alpha | M0R9L0 1xPhospho [S1190(100)] | -0.53211 | 1.659854657 |
| Dual specificity phosphatase 27, | D3ZRM0 1xPhospho [S297(99.1)] | -0.51457 | 2.641300038 |
| Vacuolar fusion protein MON1 homolog | B1WC06 2xPhospho [S56(100);S72(98.1)] | -0.51265 | 1.751682294 |
| Bromodomain containing 2 | A0A0G2K1Z8 1xPhospho [S512(100)] | -0.51457 | 2.472399679 |
| AKT1 substrate 1 | A0A8I5ZRQ9 4xPhospho [S218(100);S219(100);S227(100);S228(100)] | -0.51903 | 1.467735577 |
| Phosphatidate cytidylyltransferase 2 | Q91XU8 2xPhospho [S20(98.6);S32(100)] | -0.52763 | 2.152037734 |
| Nucleolin; Nucleolin | P13383 2xPhospho [S145(100);S157(100)];A0A8I6A236 2xPhospho [S133(100);S145(100)] | -0.51937 | 2.424279274 |
| Protein phosphatase inhibitor 2 | P50411 3xPhospho [S121(100);S122(100);S130(100)] | -0.52223 | 2.10910639 |
| Transcription initiation factor IIF subunit alpha | A0A8I6A212 1xPhospho [S207(100)] | -0.50696 | 1.961407761 |
| Glucocorticoid induced 1 | A0A8I6ACU6 3xPhospho [S92(99.4);S/T] | -0.51847 | 1.921461611 |
| A-kinase anchor protein 12 | Q5QD51 1xPhospho [S283(98.9)] | -0.50748 | 1.312497515 |
| Serine/arginine repetitive matrix 2 | A0A8I6A0A2 2xPhospho [S1317(98.6);S1322(100)] | -0.50696 | 1.495737056 |
| Nuclear casein kinase and cyclin-dependent kinase substrate 1 | A0A0G2K7X3 1xPhospho [S157(100)] | -0.50447 | 1.379618631 |
| RNA binding protein | Q5PQR0 2xPhospho [T275(100);S277(100)] | -0.49033 | 2.060881984 |
| DnaJ heat shock protein family (Hsp40) member C1 | F1LVX1 2xPhospho [S490(100);S491(100)] | -0.49341 | 1.495679372 |
| PPARGC1 and ESRR induced regulator | D3ZH76 1xPhospho [S198(100)] | -0.5025 | 1.580598966 |
| Pericentriolar material 1 | A0A8I5ZUW5 1xPhospho [S1356(100)] | -0.49185 | 1.448012248 |
| Vesicle-fusing ATPase | Q6IRG3 2xPhospho [S97(100);S99(100)] | -0.50564 | 1.728569106 |
| Glycogen [starch] synthase | A0A8I5ZWA2 3xPhospho [S588(100);S589(100);S593(100)] | -0.49304 | 1.589104488 |
| Thioredoxin domain containing 1 | Q52KJ9 1xPhospho [S245(100)] | -0.4881 | 1.46399323 |
| Eukaryotic translation elongation factor 1 delta | A0A8I5ZUU1 1xPhospho [S157(100)] | -0.50155 | 2.460199984 |
| Unconventional myosin-Ie | Q63356 1xPhospho [S1001(100)] | -0.49957 | 1.956666126 |
| Zinc finger CCCH-type containing 18 | A0A140TAG3 1xPhospho [S80(100)] | -0.49812 | 2.432664542 |
| Thymopoietin; Phosphate carrier protein | A0A8I6AFR4 3xPhospho [S155(100);S158(100);T163(100)];A0A8I6GF99 3xPhospho [S155(100);S158(100);T163(100)] | -0.50467 | 1.497477814 |
| Catenin alpha 1 | Q5U302 2xPhospho [S657(96.4);S/T] | -0.50007 | 1.30120732 |
| HECT-type E3 ubiquitin transferase | D4ADD3 1xPhospho [S1046(100)] | -0.48543 | 1.521787693 |
| Caveolae-associated protein 2 | Q66H98 1xPhospho [S362(100)] | -0.47644 | 1.920947725 |
| Eukaryotic translation initiation factor 5 | Q07205 2xPhospho [S387(100);S388(100)] | -0.47162 | 1.764139325 |
| Sequestosome-1 | O08623 1xPhospho [S354(100)] | -0.47325 | 1.511129196 |
| DNA topoisomerase 2 | A0A8I5Y7L4 1xPhospho [S1363(100)] | -0.48543 | 1.709083263 |
| Tyrosine-protein kinase HCK | P50545 1xPhospho [T410(100)] | -0.47508 | 1.819843469 |
| Methylosome subunit pICln | Q6P9X1 1xPhospho [S100(100)] | -0.48543 | 2.277600782 |
| Phosphatidate cytidylyltransferase 2 | Q91XU8 1xPhospho [S32(99)] | -0.49139 | 2.12603761 |
| Titin; Cardiac titin N2B isoform; Cardiac titin fetal N2BA PEVK isoform | A0A8I5ZUN3 1xPhospho [S12982(100)];Q7TMZ9 1xPhospho [S1192(100)];Q5VJM4 1xPhospho [S1019(100)] | -0.48543 | 1.4562882 |
| Ral GTPase activating protein catalytic subunit alpha 1 | A0A8I6A9F7 1xPhospho [T753(100)] | -0.47482 | 2.965678428 |
| Microtubule-associated protein 1A | G3V7U2 1xPhospho [S1622(99.1)] | -0.47468 | 1.725704968 |
| Myosin heavy chain 7; Myosin heavy chain 2; Myosin heavy chain 6 | G3V8B0 1xPhospho [S1199(100)];G3V6E1 1xPhospho [S1206(100)];G3V885 1xPhospho [S1201(100)] | -0.46632 | 2.057344611 |
| Fam134c protein | B2GV94 3xPhospho [T310(100);S313(100);S320(100)] | -0.47534 | 2.669563537 |
| Thioredoxin domain containing 1 | Q52KJ9 1xPhospho [S245(100)] | -0.46113 | 3.434907539 |
| Pyridoxal-dependent decarboxylase domain | A0A1W2Q6L1 1xPhospho [S752(100)] | -0.46815 | 2.804704634 |
| CD2-associated protein | F1LRS8 1xPhospho [S458(100)] | -0.46424 | 2.013165268 |
| Eukaryotic translation elongation factor 1 delta | A0A8I5ZUU1 1xPhospho [S157(100)] | -0.46395 | 2.138911624 |
| Uncharacterized LOC108351606 | A0A0G2JXY3 1xPhospho [S147(99.3)] | -0.45686 | 3.085255801 |
| Uncharacterized LOC108351606 | A0A0G2JXY3 2xPhospho [S147(100);T149(100)] | -0.47454 | 1.957535845 |
| Phosphatidate cytidylyltransferase 2 | Q91XU8 1xPhospho [S32(100)] | -0.45768 | 2.680230979 |
| Mitochondrial dynamics protein MID51 | Q5XIS8 2xPhospho [S55(100);S59(99.2)] | -0.46877 | 1.766099534 |
| Tumor protein p53-inducible protein 11 | B3DMA0 1xPhospho [S14(100)] | -0.45638 | 1.674871308 |
| SWI/SNF-related matrix-associated actin-dependent regulator of chromatin subfamily A-containing DEAD/H box 1 | A0A8I6APK9 2xPhospho [S80(100);S83(100)] | -0.46073 | 2.561713357 |
| Nascent polypeptide associated complex | M0R9L0 1xPhospho [S1362(100)] | -0.45138 | 1.340964266 |
| Voltage-dependent anion channel 1 | A0A8I6AKF7 1xPhospho [S259(100)] | -0.44652 | 2.370224512 |
| Nexilin (F actin binding protein) | A0A8I5ZUL4 1xPhospho [S159(100)] | -0.44931 | 1.622988157 |
| Actin binding LIM protein family | F1M8U2 2xPhospho [S386(98.8);S401(98.8)] | -0.4634 | 1.381777356 |
| Striated muscle enriched protein kinase | A0A8I6ADG2 3xPhospho [T379(100);S382(100);S385(100)] | -0.45457 | 2.132434928 |
| Coiled-coil domain-containing protein 43 | Q5BK07 1xPhospho [T137(100)] | -0.46211 | 1.901597465 |
| Tight junction protein ZO-1 | A0A0G2K2P5 1xPhospho [S125(100)] | -0.45251 | 2.219416497 |
| Nuclear export mediator factor | A0A8I6A9M7 3xPhospho [S732(100);S738(100);S739(100)] | -0.45372 | 1.489732453 |
| Ribosomal protein S6 kinase C1 | A0A8I6GH44 1xPhospho [S281(99.3)] | -0.45638 | 1.402344591 |
| Glycogen [starch] synthase | A0A8I5ZWA2 2xPhospho [S588(100);S589(100)] | -0.45111 | 1.483068344 |
| GIT ArfGAP 1 | A0A8I6AGH2 2xPhospho [S378(100);S381(100)] | -0.43472 | 1.905656837 |
| Nuclear factor 1 | A0A8I5ZTI5 2xPhospho [S289(99);S300(100)] | -0.44746 | 1.731367824 |
| 3-phosphoinositide-dependent protein kinase 1 | O55173 1xPhospho [S244(100)] | -0.44313 | 2.208779226 |
| Phosphotyrosine interaction domain containing 1 | A0A8I6GLY4 1xPhospho [S200(100)] | -0.44876 | 1.703680165 |
| Matrix remodeling associated 7 | A0A8I6GB26 1xPhospho [S89(100)] | -0.43172 | 1.990217878 |
| Pleckstrin homology-like domain; Pleckstrin homology-like domain | A0A8I6A9F1 2xPhospho [S696(99.2);S/T];A0A0G2JV32 2xPhospho [S696(99.2);S/T] | -0.4361 | 2.47830262 |
| Membrane-associated progesterone receptor component 1 | P70580 1xPhospho [S181(100)] | -0.45606 | 2.124177137 |
| Poly(A) binding protein | A0A8I5ZSJ2 1xPhospho [S199(100)] | -0.44668 | 2.150811351 |
| AHNAK nucleoprotein; AHNAK nucleoprotein | A0A0G2JU96 1xPhospho [S5221(100)];A0A0G2JUA5 1xPhospho [S5349(100)] | -0.44441 | 2.546099622 |
| TBC1 domain family, member 2B | A0A8I5Y6N8 1xPhospho [S860(98.7)] | -0.43741 | 1.931823739 |
| Myosin heavy chain 14 | A0A8I6A9A3 1xPhospho [S1920(100)] | -0.43929 | 1.40406818 |
| Vimentin | P31000 1xPhospho [S325(100)] | -0.43063 | 2.371656471 |
| Thioredoxin domain containing 1 | Q52KJ9 1xPhospho [S245(100)] | -0.42833 | 2.399445689 |
| Glycogen [starch] synthase | A0A8I5ZWA2 3xPhospho [S577(100);S581(100);S585(100)] | -0.44323 | 1.369636805 |
| Pseudopodium-enriched atypical kinase 1 | D4A563 1xPhospho [S281(100)] | -0.4344 | 2.402242695 |
| Transcription factor 4 | Q62655 1xPhospho [S433(98.9)] | -0.4344 | 2.954877583 |
| Bcl-2-interacting death suppressor;BAG cochaperone 3 | Q156J1 1xPhospho [S138(100)];Q5U2U8 1xPhospho [S138(100)] | -0.43252 | 1.354757794 |
| Sorting nexin 29 | A0A8I6A5K5 1xPhospho [S268(100)] | -0.42475 | 1.74289033 |
| MICAL-like 1 | A0A8I5YBZ3 1xPhospho [S492(100)] | -0.42884 | 2.269882817 |
| Striated muscle enriched protein kinase | A0A8I6ADG2 1xPhospho [S2137(100)] | -0.41504 | 1.458865438 |
| Kelch-like family member 31 | D3ZLT6 3xPhospho [S626(99.3);S628(99.3);S633(100)] | -0.41923 | 1.788919032 |
| Eukaryotic translation initiation factor 2 subunit beta | Q6P685 1xPhospho [S105(100)] | -0.4245 | 2.56182557 |
| Heterogeneous nuclear ribonucleoprotein L-like | D4A3E1 1xPhospho [S94(100)] | -0.4235 | 1.318278734 |
| Nuclear casein kinase and cyclin-dependent kinase substrate 1 | A0A0G2K7X3 3xPhospho [S273(100);S278(100);S284(100)] | -0.42576 | 2.396511892 |
| Phosducin-like protein 3 | Q4KLJ8 1xPhospho [S65(100)] | -0.41172 | 2.367094111 |
| Microtubule-associated protein 1A | G3V7U2 1xPhospho [S666(100)] | -0.4112 | 2.418880697 |
| Anthrax toxin receptor 1 | Q0PMD2 1xPhospho [S360(100)] | -0.40788 | 2.635640642 |
| RPTOR independent companion of MTOR, complex 2 | A0A8I5ZNG4 1xPhospho [S21(100)] | -0.41181 | 2.300223656 |
| Dual specificity phosphatase 27 | D3ZRM0 1xPhospho [S552(100)] | -0.41504 | 2.625046694 |
| Unc-119 lipid binding chaperone | A0A8I6ARX8 2xPhospho [S37(100);S39(100)] | -0.41028 | 2.135964665 |
| Heat shock protein HSP 90-alpha | P82995 1xPhospho [S263(100)] | -0.42414 | 1.871918965 |
| Eukaryotic elongation factor 2 kinase | Q6P757 1xPhospho [S444(100)] | -0.40244 | 1.557369617 |
| Ankyrin repeat and sterile alpha motif domain containing 1A | D4AC12 1xPhospho [S641(98.9)] | -0.41143 | 1.303189583 |
| Rho GTPase activating protein 5 | A0A0G2K7N9 1xPhospho [S1174(99.6)] | -0.40255 | 2.844331607 |
| Calnexin | P35565 3xPhospho [S553(100);T561(100);S563(100)] | -0.41504 | 2.440415191 |
| EH domain binding protein 1 | A0A8I5ZZB4 1xPhospho [S897(100)] | -0.41504 | 1.52170103 |
| SIN3 transcription regulator family member A | A0A0G2K3H5 1xPhospho [S816(100)] | -0.40407 | 2.18089472 |
| Serine/threonine-protein kinase TAO3 | Q53UA7 1xPhospho [S324(100)] | -0.39855 | 1.723359531 |
| DENN domain containing 1B | M0R8I4 1xPhospho [S141(100)] | -0.38957 | 1.497841764 |
| Protein phosphatase inhibitor 2 | P50411 1xPhospho [S122(100)] | -0.39463 | 3.638023395 |
| Protein phosphatase 1 regulatory subunit 14A | Q99MC0 1xPhospho [S26(100)] | -0.39541 | 1.58485849 |
| Type 2X myosin heavy chain | Q9QZV8 1xPhospho [S136(100)] | -0.40663 | 1.367641778 |
| Cytochrome c oxidase subunit 4 | A0A8I5ZUV1 1xPhospho [S80(100)] | -0.40276 | 1.497605952 |
| Elongation factor 1-beta | B5DEN5 1xPhospho [S106(100)] | -0.39079 | 2.126933501 |
| Tight junction protein ZO-1 | A0A0G2K2P5 2xPhospho [S125(100);S131(100)] | -0.41079 | 1.865217626 |
| Sodium channel protein type 5 subunit alpha | P15389 2xPhospho [S458(99);S/T] | -0.39855 | 1.715897185 |
| Holliday junction recognition protein | D4A1W1 1xPhospho [S667(98.2)] | -0.39514 | 2.287267367 |
| E3 ubiquitin-protein ligase | B4F767 1xPhospho [S211(100)] | -0.38904 | 1.711550341 |
| Outer mitochondrial transmembrane helix translocase | Q505J9 1xPhospho [S322(100)] | -0.38333 | 1.796018881 |
| MARCKS-related protein | Q9EPH2 2xPhospho [S132(100);S135(100)] | -0.38773 | 1.787916013 |
| Striatin interacting protein 2 | A0A8I5ZP29 1xPhospho [S364(100)] | -0.38904 | 2.32642781 |
| C2CD2-like | F7EUW3 2xPhospho [S625(98.1);S/T] | -0.38618 | 1.40127073 |
| Bcl-2-interacting death suppressor | Q156J1 1xPhospho [S401(100)] | -0.39652 | 1.972847932 |
| Myocyte enhancer factor 2D | A0A8I6GLN1 1xPhospho [S97(100)] | -0.38782 | 2.330237697 |
| Casein kinase I isoform alpha | P97633 1xPhospho [T321(95.6)] | -0.39102 | 2.030497593 |
| Member RAS oncogene family | D4A376 1xPhospho [S105(100)] | -0.38904 | 3.11059639 |
| Heat shock protein family A (Hsp70) member 4 like | F7F2F3 1xPhospho [S253(100)] | -0.37851 | 1.41988191 |
| Pre-mRNA 3'-end-processing factor FIP1 | A0A8I5ZW26 1xPhospho [S532(100)] | -0.38904 | 1.344120511 |
| Nitric oxide synthase | A0A8I6A2Y1 1xPhospho [S1176(100)] | -0.39666 | 2.961213875 |
| Endonuclease/exonuclease/phosphatase family domain-containing protein 1 | A0A8I5ZPH9 2xPhospho [S106(100);S] | -0.40296 | 1.684251105 |
| Rho GTPase activating protein 31 | D4A987 2xPhospho [T615(100);S619(100)] | -0.38782 | 3.844498599 |
| Epidermal growth factor receptor pathway substrate 15-like 1 | A0A8I5ZKJ0 1xPhospho [S219(100)] | -0.38702 | 1.554794995 |
| DNA topoisomerase 2 | A0A8I5Y7L4 1xPhospho [S1570(100)] | -0.38529 | 2.206263093 |
| Membrane-associated progesterone receptor component 1 | P70580 1xPhospho [S181(100)] | -0.39046 | 2.052203947 |
| Sh3bgr protein | B0K040 1xPhospho [S182(100)] | -0.39046 | 1.583330548 |
| Vesicle-fusing ATPase | F7EZ84 1xPhospho [S76(100)] | -0.38109 | 3.129533776 |
| LRR binding FLII interacting protein 2 | A0A8I5ZUL9 1xPhospho [S355(100)] | -0.38082 | 1.538532182 |
| Thymopoietin; Phosphate carrier protein | A0A8I6AFR4 3xPhospho [S66(100);S67(100);T74(100)];A0A8I6GF99 3xPhospho [S66(100);S67(100);T74(100)] | -0.40407 | 1.998688507 |
| Zinc finger CCCH-type containing 18 | A0A140TAG3 2xPhospho [S127(100);T/S] | -0.37851 | 2.551530433 |
| ATP-dependent 6-phosphofructokinase | Q52KS1 3xPhospho [T636(94.6);T637(94.6);Y644(91.8)] | -0.37851 | 1.423289042 |
| Protein phosphatase inhibitor 2 | P50411 2xPhospho [S121(100);S122(100)] | -0.37126 | 3.388242927 |
| G3BP stress granule assembly factor 1 | A0A8I6A800 1xPhospho [S105(100)] | -0.38109 | 2.165767842 |
| DNA topoisomerase 2 | A0A8I5Y7L4 1xPhospho [S1541(100)] | -0.37137 | 1.93847462 |
| Non-specific serine/threonine protein kinase | A0A8I6A6A2 1xPhospho [S649(100)] | -0.36965 | 1.67976369 |
| ATP-binding cassette sub-family C member 9 | Q63563 1xPhospho [S964(98.8)] | -0.37551 | 1.827829801 |
| Signal-induced proliferation-associated 1 like 3 | F1LYG2 1xPhospho [S94(98.7)] | -0.36804 | 1.834278693 |
| Pleckstrin homology and FYVE domain containing 2 | B1WBV4 1xPhospho [S248(99)] | -0.38143 | 1.587057063 |
| Glycogen [starch] synthase | A0A8I5ZWA2 2xPhospho [S577(100);S581(100)] | -0.38272 | 2.07180042 |
| AHNAK nucleoprotein; AHNAK nucleoprotein | A0A0G2JU96 1xPhospho [S5221(100)];A0A0G2JUA5 1xPhospho [S5349(100)] | -0.38125 | 2.206816496 |
| Actin-binding LIM protein 1; Actin-binding LIM protein 1 | A0A8I6A592 1xPhospho [S622(100)];A0A8I6G9J9 1xPhospho [S543(100)] | -0.38333 | 1.942644029 |
| Thioredoxin domain containing 1 | Q52KJ9 1xPhospho [S245(100)] | -0.37587 | 1.941941262 |
| cAMP-dependent protein kinase type II-alpha | P12368 1xPhospho [S97(100)] | -0.37197 | 2.581689257 |
| RAN binding protein 3 | M0R5Q3 2xPhospho [S32(96.9);S40(100)] | -0.38187 | 1.347685987 |
| Nuclear export mediator factor | A0A8I6A9M7 2xPhospho [S738(98.9);S739(98.9)] | -0.37126 | 1.455442303 |
| Ankyrin-3 | A0A0G2K1Q7 1xPhospho [S1658(100)] | -0.37126 | 2.628714067 |
| Centrosomal protein 170 | A0A0G2K315 2xPhospho [S253(98.7);S256(100)] | -0.38156 | 1.422562102 |
| O-GlcNAcase | A0A8I6AAH5 1xPhospho [S396(100)] | -0.36257 | 2.33432575 |
| Non-specific serine/threonine protein kinase | A0A8I5Y708 1xPhospho [S295(100)] | -0.36441 | 3.30296884 |
| RAB11 binding and LisH domain | A0A8I5ZRA1 1xPhospho [S180(100)] | -0.37346 | 1.77164376 |
| PDZ and LIM domain 4 | M0R4H5 1xPhospho [S124(100)] | -0.37304 | 1.314056882 |
| Exocyst complex component 1 | A0A8I6A8D1 1xPhospho [S504(100)] | -0.36976 | 2.127304542 |
| Arf-GAP domain and FG repeat-containing protein 1 | Q4KLH5 1xPhospho [S181(100)] | -0.37493 | 1.545089371 |
| Coiled-coil-helix-coiled-coil-helix domain containing 2 | Q5BJB3 1xPhospho [S46(100)] | -0.35693 | 1.861489556 |
| Nexilin; Nexilin (F actin binding protein) | C5H4P9 2xPhospho [S339(100);S344(98.9)];A0A8I5ZUL4 2xPhospho [S558(100);S563(98.9)] | -0.37064 | 1.337877877 |
| Catenin alpha 1 | Q5U302 2xPhospho [S643(100);S654(100)] | -0.37724 | 2.80316769 |
| Myosin heavy chain 7 | G3V8B0 1xPhospho [T1019(100)] | -0.37197 | 1.740835118 |
| Glutamate receptor-interacting protein 2 | Q9WTW1 1xPhospho [S646(100)] | -0.37397 | 1.390995284 |
| SWI/SNF-related matrix-associated actin-dependent regulator of chromatin a2 | Q6DUH4 3xPhospho [S1519(100);S1523(100);S1535(100)] | -0.36513 | 1.712827944 |
| RCG37353, isoform CRA_b | Q5PQR0 3xPhospho [T275(97.3);S284(100);T287(100)] | -0.34933 | 1.499500403 |
| Optineurin | Q2MH90 1xPhospho [S181(100)] | -0.35755 | 1.634849462 |
| Filamin A interacting protein 1-like | D4A900 2xPhospho [T984(100);T992(100)] | -0.36388 | 1.58207456 |
| Eukaryotic translation elongation factor 1 delta | A0A8I5ZUU1 2xPhospho [T142(100);S157(100)] | -0.34994 | 1.540686782 |
| Nucleosome assembly protein 1-like 4 | A0A8I6G1Z9 1xPhospho [S125(100)] | -0.34577 | 1.876772022 |
| N-terminal amino-acid N(alpha)-acetyltransferase NatA | D3ZUQ2 2xPhospho [S213(98.7);S/T] | -0.361 | 1.515671182 |
| Xin actin-binding repeat containing 1 | A0A8I5YBK9 1xPhospho [S331(100)] | -0.36613 | 1.943981304 |
| Oxysterol-binding protein | D4A9D8 2xPhospho [S188(100);S191(100)] | -0.37126 | 2.296136226 |
| AKT1 substrate 1 | A0A8I5ZRQ9 3xPhospho [S219(98.8);S227(100);S228(100)] | -0.36088 | 2.573359712 |
| Catenin alpha 3 | A0A0G2JX81 3xPhospho [S656(100);S666(98.6);S669(97.2)] | -0.35669 | 1.506004629 |
| Elongation factor 1-beta | B5DEN5 1xPhospho [S106(100)] | -0.35117 | 1.673777835 |
| Protein phosphatase inhibitor 2 | P50411 3xPhospho [S121(100);S122(100);S130(100)] | -0.35566 | 2.289446879 |
| Ubiquitin protein ligase E3 component n-recognin 4 | A0A0G2JU89 1xPhospho [T2735(100)] | -0.36075 | 1.946370918 |
| Zinc finger CCCH type containing 13 | E9PSN4 2xPhospho [S1088(100);S1091(100)] | -0.34792 | 1.326879548 |
| EMAP like 1 | A0A8I6A157 1xPhospho [S126(100)] | -0.35657 | 1.326690862 |
| Synembryn | B1H241 1xPhospho [S435(100)] | -0.34915 | 2.211539724 |
| Lysine-specific histone demethylase | B3STT9 2xPhospho [S131(100);S137(100)] | -0.35184 | 1.914163741 |
| ATP-binding cassette sub-family F member 1 | Q6MG08 1xPhospho [S109(100)] | -0.34395 | 2.502700124 |
| RAF proto-oncogene serine/threonine-protein kinase | P11345 1xPhospho [S259(98.7)] | -0.36257 | 1.470800982 |
| Synembryn | A0A8I5Y0A0 2xPhospho [S468(100);T473(99.2)] | -0.33703 | 3.270429561 |
| Zinc finger Ran-binding domain-containing protein 2 | A0A8I6ASM5 1xPhospho [S116(100)] | -0.34008 | 1.405972464 |
| Torsin-1A-interacting protein 1 | Q5PQX1 1xPhospho [S157(99.3)] | -0.35311 | 1.781422168 |
| Striated muscle enriched protein kinase | A0A8I6ADG2 1xPhospho [S2044(100)] | -0.33921 | 1.723210866 |
| Protein kinase C | A0A8I6G981 1xPhospho [S382(100)] | -0.33703 | 1.60238141 |
| Zinc finger (CCCH type) | Q5RK33 1xPhospho [S51(100)] | -0.3377 | 1.744312873 |
| Cell cycle and apoptosis regulator 2 | F1LM55 1xPhospho [S677(100)] | -0.33703 | 1.432280181 |
| Microfibrillar-associated protein 1A | D4ACM9 2xPhospho [S52(100);S53(100)] | -0.34104 | 1.844584794 |
| Pericentriolar material 1 | A0A8I5ZUW5 2xPhospho [S1868(100);S1871(100)] | -0.32193 | 1.41666885 |
| Myozenin 2 | A0A0G2KAQ5 2xPhospho [S116(98.3);S/T/Y] | -0.3254 | 2.188554351 |
| Regulator of cell cycle | A0A8I6AG65 2xPhospho [S83(100);S/Y] | -0.33498 | 1.583129736 |
| Remodeling and spacing factor 1 | D3ZGQ8 3xPhospho [T1313(100);S1317(100);S1320(100)] | -0.32524 | 1.550234834 |
| Large ribosomal subunit protein uL10 | P19945 2xPhospho [S304(100);S307(100)] | -0.3339 | 1.439165772 |
| DEAD-box helicase 42 | D4A031 1xPhospho [S185(98.4)] | -0.32957 | 2.195870749 |
| GTP-binding protein 1 | D2XV59 2xPhospho [S25(98.3);S] | -0.32496 | 1.33606273 |
| Protein PRRC2A | Q6MG48 2xPhospho [S342(100);S350(100)] | -0.32193 | 1.794097784 |
| Calnexin | P35565 2xPhospho [S553(100);S563(99.2)] | -0.33787 | 1.986122885 |
| Synergin | A0A8I5ZJM5 1xPhospho [S775(100)] | -0.34104 | 2.458619662 |
| Ataxin 2 | A0A8I5ZRB2 1xPhospho [S395(100)] | -0.34049 | 1.725525726 |
| PDZ and LIM domain 5; PDZ and LIM domain 5 | A0A8I6GJY3 1xPhospho [S119(100)];A0A8I6G7D0 1xPhospho [S119(100)] | -0.32681 | 1.680274154 |
| MAGE family member D2 | Q3B7U1 2xPhospho [S191(100);S194(100)] | -0.32443 | 1.358231865 |
| O-GlcNAcase | A0A8I6AAH5 1xPhospho [S396(100)] | -0.31895 | 1.642459049 |
| RCG37353, isoform CRA_b | Q5PQR0 1xPhospho [T275(98.7)] | -0.31882 | 1.57508901 |
| Tight junction protein ZO-1 | A0A0G2K2P5 2xPhospho [S125(100);S131(100)] | -0.32985 | 1.773730258 |
| Mbd2 protein | B0BNM2 1xPhospho [S408(100)] | -0.32862 | 1.690732332 |
| 5'-3' exoribonuclease | A0A8I6A511 2xPhospho [S463(100);S465(100)] | -0.32665 | 1.694221852 |
| Zinc finger protein 830 | Q3MHS2 1xPhospho [S349(100)] | -0.32462 | 1.327348361 |
| RCG32667 | A6HIS0 1xPhospho [S161(99.5)] | -0.33374 | 2.56688421 |
| LIM domain and actin binding 1 | A0A8I6AM55 1xPhospho [S243(100)] | -0.32193 | 1.585452459 |
| Histone deacetylase 2 | F7ENH8 1xPhospho [S422(100)] | -0.32524 | 1.669689294 |
| Ubiquitin protein ligase E3 component n-recognin 4 | A0A0G2JU89 1xPhospho [T2735(100)] | -0.3251 | 3.074638259 |
| LIM and calponin homology domains 1 | F1M392 2xPhospho [T215(100);S217(100)] | -0.32906 | 1.327458909 |
| Family with sequence similarity 98 | A0A8I6AK78 1xPhospho [S43(100)] | -0.30875 | 2.121225156 |
| Multivesicular body subunit 12A | A0A8I6AIU5 1xPhospho [S168(100)] | -0.31375 | 1.615321027 |
| Microfibrillar-associated protein 1A | D4ACM9 2xPhospho [S132(100);S133(100)] | -0.3278 | 1.307363662 |
| Interacts with SUPT6H | A0A8I6AQ12 1xPhospho [S328(100)] | -0.30563 | 1.430674936 |
| Catenin alpha 3 | A0A0G2JX81 4xPhospho [S656(97.4);S666(97.4);S669(99.9);T670(99.8)] | -0.31797 | 1.304932633 |
| Connector enhancer of kinase suppressor of Ras 1 | G3V8W8 2xPhospho [S535(100);T537(100)] | -0.30563 | 1.63194615 |
| Uncharacterized protein C11orf96 homolog | A8IHN8 1xPhospho [S192(100)] | -0.31279 | 1.777518736 |
| Serine/threonine-protein kinase 3 | O54748 1xPhospho [S316(100)] | -0.31238 | 3.051428512 |
| FUN14 domain-containing protein 1 | Q5BJS4 1xPhospho [S13(100)] | -0.3254 | 1.704125328 |
| Eukaryotic translation initiation factor 3 subunit B | Q4G061 2xPhospho [S75(100);S79(98.9)] | -0.3135 | 2.273901613 |
| Tetratricopeptide repeat | A0A8I6A9F6 2xPhospho [S1451(99.6);T/S] | -0.31034 | 1.782430689 |
| Family with sequence similarity 219, member A | A0A8I6GG65 1xPhospho [S114(100)] | -0.30666 | 1.973823128 |
| Electrogenic sodium bicarbonate cotransporter 1 | Q9JI66 1xPhospho [S1029(100)] | -0.31895 | 1.551035338 |
| RAB, member RAS oncogene family-like 6 | D3ZKQ4 2xPhospho [S489(100);S494(100)] | -0.31451 | 1.81892946 |
| Atrophin 1 | G3V7W3 2xPhospho [S100(100);S106(100)] | -0.31487 | 2.306432615 |
| Rho guanine nucleotide exchange factor 1 | A0A0G2JU01 1xPhospho [T432(100)] | -0.31487 | 3.209838259 |
| MAP7 domain containing 1 | A0A8I5ZM56 1xPhospho [S503(100)] | -0.3152 | 1.446606859 |
| Titin | A0A8I6A794 3xPhospho [S4935(98.7);T4936(98.7);S/T/Y];3xPhospho [S4957(98.7);T4958(98.7);S/T/Y] | -0.31382 | 1.893469288 |
| MAGE family member D2 | Q3B7U1 3xPhospho [S191(100);S194(98.9);S197(97.8)] | -0.30307 | 1.305914314 |
| WAPL cohesin release factor | D4ADT3 1xPhospho [S77(99.3)] | -0.29501 | 1.329673593 |
| Ankyrin-3 | A0A0G2K4D0 1xPhospho [S830(100)] | -0.30066 | 1.369507532 |
| Mbd2 protein | B0BNM2 1xPhospho [S408(100)] | -0.29598 | 2.400417492 |
| Very-long-chain (3R)-3-hydroxyacyl-CoA dehydratase | D4ABI7 1xPhospho [S114(100)] | -0.2949 | 3.466111611 |
| ATP-binding cassette sub-family F member 1 | Q6MG08 2xPhospho [T195(100);S197(100)] | -0.28951 | 2.60034363 |
| Zinc finger protein 592 | D3ZJG8 1xPhospho [S1200(100)] | -0.29255 | 1.847981005 |
| Eukaryotic translation initiation factor 4 gamma, 3 | A0A0G2JY73 1xPhospho [S526(100)] | -0.30875 | 4.012669748 |
| BLOC-1-related complex subunit 6 | Q66H43 1xPhospho [S130(100)] | -0.29662 | 2.040703535 |
| DNA topoisomerase 2 | A0A8I5Y7L4 1xPhospho [S1453(100)] | -0.30485 | 1.970166019 |
| Protein phosphatase 1 regulatory subunit | A0A8I6A6B6 1xPhospho [S773(99.4)] | -0.30051 | 1.652793759 |
| Phosphoribosyl pyrophosphate synthase-associated | O08618 1xPhospho [S227(100)] | -0.29662 | 2.656840478 |
| La ribonucleoprotein 1 | A0A8I6GJC3 2xPhospho [S605(100);S609(100)] | -0.30051 | 2.187888253 |
| Pcmtd2 protein | B5DF20 2xPhospho [S75(100);S78(100)] | -0.30415 | 1.53910955 |
| Vps35 protein | B5DFC1 1xPhospho [S616(100)] | -0.30339 | 1.471321542 |
| Spectrin beta chain | Q6XD99 4xPhospho [T2153(99.3);S2159(98.6);T2165(97.2);S] | -0.30339 | 1.452891569 |
| Choline transporter-like protein | A0A8I6G961 1xPhospho [S698(99.1)] | -0.30339 | 1.461759112 |
| Cytosolic phospholipase A2 | P50393 1xPhospho [S435(98.9)] | -0.29445 | 2.071648363 |
| PBX homeobox interacting protein 1 | A0A8I6GLZ5 3xPhospho [S156(100);S157(100);S158(100)] | -0.29418 | 1.4470968 |
| Oxysterol-binding protein | D4A9D8 2xPhospho [S188(100);S191(100)] | -0.30256 | 2.095490258 |
| Histone deacetylase 2 | F7ENH8 2xPhospho [S422(100);S424(100)] | -0.28662 | 1.883064239 |
| ATP-dependent RNA helicase | Q6VEU8 1xPhospho [S80(100)] | -0.2854 | 2.09499393 |
| Phosphatidate cytidylyltransferase 2 | Q91XU8 1xPhospho [S32(100)] | -0.30256 | 1.684334402 |
| Ubiquitin carboxyl-terminal hydrolase | A0A8I6AC85 2xPhospho [S132(100);S134(100)] | -0.27753 | 1.824427102 |
| Translocon-associated protein subunit alpha | A0A8I5ZVE2 1xPhospho [S256(100)] | -0.28951 | 1.7437664 |
| HIV-1 Tat specific factor 1 | D4A997 1xPhospho [S743(100)] | -0.27898 | 1.388425594 |
| Thymopoietin; Phosphate carrier protein | A0A8I6AFR4 2xPhospho [S66(100);S67(100)];A0A8I6GF99 2xPhospho [S66(100);S67(100)] | -0.28813 | 1.373383557 |
| Unc-119 lipid binding chaperone | A0A8I6ARX8 1xPhospho [S37(100)] | -0.28118 | 2.029989226 |
| Nexilin (F actin binding protein) | A0A8I5ZUL4 1xPhospho [S80(100)] | -0.29218 | 2.055264373 |
| Taxilin beta | A0A0G2K2T1 2xPhospho [T481(100);S482(100)] | -0.29474 | 3.355717916 |
| RAN binding protein 3 | M0R5Q3 1xPhospho [S40(100)] | -0.28118 | 1.46681204 |
| AHNAK nucleoprotein; AHNAK nucleoprotein | A0A0G2JU96 1xPhospho [S5273(100)];A0A0G2JUA5 1xPhospho [S5401(100)] | -0.28567 | 1.451668712 |
| Nexilin; Nexilin (F actin binding protein) | C5H4P9 3xPhospho [S339(100);T346(98.7);S/T];A0A8I5ZUL4 3xPhospho [S558(100);T565(98.7);S/T] | -0.28473 | 1.570872063 |
| 2-oxoisovalerate dehydrogenase subunit alpha | A0A8I6GGA5 3xPhospho [S334(94.3);T335(94.3);S/Y] | -0.29834 | 1.304023805 |
| Titin; Titin | A0A8I5ZUN3 2xPhospho [S1423(100);S1428(100)];A0A8I6A794 2xPhospho [S1378(100);S1383(100)] | -0.28652 | 2.362192058 |
| Insulin-like growth factor 2 receptor | G3V824 1xPhospho [S2399(100)] | -0.27684 | 2.930110485 |
| WASH complex subunit 2C; WASH complex subunit 2C | F1LPG9 1xPhospho [S525(99.5)];A0A8I6AM40 1xPhospho [S577(99.5)] | -0.28473 | 1.945974433 |
| Kruppel like factor 3 | D4A4V3 1xPhospho [S71(100)] | -0.28011 | 2.553253119 |
| Cdc42 effector protein 2 | Q5PQP4 1xPhospho [S137(100)] | -0.26303 | 1.768706311 |
| Bystin | Q80WL2 1xPhospho [S97(100)] | -0.2783 | 1.545795036 |
| PC4 and SFRS1 interacting protein 1 | A0A0G2JTA2 2xPhospho [T271(98.6);S272(98.6)] | -0.27009 | 1.463004175 |
| E3 ubiquitin-protein ligase TRIP12 | F1LP64 2xPhospho [S1409(100);T1410(100)] | -0.26303 | 2.443311215 |
| Titin | A0A8I5ZUN3 1xPhospho [S29712(99.5)] | -0.26946 | 1.309570789 |
| Protein phosphatase inhibitor 2 | P50411 2xPhospho [S121(100);S122(100)] | -0.27854 | 1.600208471 |
| Cell cycle and apoptosis regulator 2 | F1LM55 2xPhospho [S674(100);S677(100)] | -0.27938 | 1.867344612 |
| Fructose-bisphosphate aldolase A | P05065 1xPhospho [S46(100)] | -0.2713 | 3.245948051 |
| G3BP stress granule assembly factor 2 | F7F5P9 2xPhospho [S225(100);T227(100)] | -0.28204 | 1.822632195 |
| Treacle ribosome biogenesis factor 1 | D4A206 1xPhospho [S1340(100)] | -0.27417 | 1.324265124 |
| Pleckstrin homology domain interacting protein | F1M3B3 2xPhospho [S1117(100);S1119(100)] | -0.28379 | 2.252345833 |
