# Supplementary material for: Ischemic preconditioning affects phosphosites and accentuates myocardial stunning while reducing infarction size in rats
Source: Front Cardiovasc Med. 2024 Mar 15;11:1376367. doi: 10.3389/fcvm.2024.1376367 (PMC10978780; doi:10.3389/fcvm.2024.1376367)
Supplement: Supplementary file 3 [file Table3.docx]

| **Cellular components** | | | | | |
| --- | --- | --- | --- | --- | --- |
| Enrichment FDR | nPhosphoproteins | Pathway Proteins | Fold Enrichment | Pathway | Proteins |
| 3.46E-10 | 14 | 87 | 13.824169 | Z disc | MYPN FLNC CRYAB ANK2 MYOZ2 MYH7 SYNPO BAG3 MYH6 NEBL RGD1564899 ALDOA OBSCN LDB3 |
| 3.35E-08 | 11 | 70 | 13.49972421 | Actin filament bundle | LIMCH1 CRYAB MYH7 SYNPO ABLIM3 BAG3 SIPA1L3 MYH6 NEBL PDLIM4 LDB3 |
| 4.14E-10 | 14 | 94 | 12.7947096 | I band | MYPN FLNC CRYAB ANK2 MYOZ2 MYH7 SYNPO BAG3 MYH6 NEBL RGD1564899 ALDOA OBSCN LDB3 |
| 4.14E-10 | 16 | 136 | 10.1067454 | Sarcomere | MYPN FLNC CRYAB ANK2 MYOZ2 MYH7 SYNPO BAG3 MYH6 FHOD3 NEBL MYH4 RGD1564899 ALDOA OBSCN LDB3 |
| 3.46E-10 | 18 | 164 | 9.428853941 | Contractile fiber | MYPN FLNC CRYAB ANK2 MYOZ2 MYH7 SVIL SYNPO BAG3 PLEC MYH6 FHOD3 NEBL MYH4 RGD1564899 ALDOA OBSCN LDB3 |
| 4.14E-10 | 17 | 155 | 9.422094906 | Myofibril | MYPN FLNC CRYAB ANK2 MYOZ2 MYH7 SVIL SYNPO BAG3 MYH6 FHOD3 NEBL MYH4 RGD1564899 ALDOA OBSCN LDB3 |
| 3.98E-08 | 29 | 654 | 3.809346699 | Supramolecular fiber | MYPN DNM1L SPECC1 MAPT FLNC DYNC1LI1 CRYAB ANK2 CD2AP MAP1A PPIP5K1 MYOZ2 MYH7 SVIL SYNPO LMNA EML3 BAG3 PLEC MYH6 FHOD3 NEBL MYH4 RGD1564899 PDLIM4 ALDOA EHBP1L1 OBSCN LDB3 |
| 4.57E-08 | 29 | 661 | 3.76900566 | Supramolecular polymer | MYPN DNM1L SPECC1 MAPT FLNC DYNC1LI1 CRYAB ANK2 CD2AP MAP1A PPIP5K1 MYOZ2 MYH7 SVIL SYNPO LMNA EML3 BAG3 PLEC MYH6 FHOD3 NEBL MYH4 RGD1564899 PDLIM4 ALDOA EHBP1L1 OBSCN LDB3 |
| 3.98E-08 | 35 | 921 | 3.264665317 | Supramolecular complex | MYPN DNM1L SPECC1 SQSTM1 GRSF1 MAPT FLNC DYNC1LI1 CRYAB ANK2 CD2AP MAP1A PPIP5K1 MYOZ2 PHF2 MYH7 SVIL SYNPO LMNA EML3 BAG3 PLEC MYH6 FHOD3 SIN3A MCRIP1 NEBL MYH4 RGD1564899 PDLIM4 ALDOA EHBP1L1 OBSCN ZFP36 LDB3 |
| 4.14E-10 | 56 | 1771 | 2.716437499 | Cytoskeleton | DNM1L LIMCH1 SPECC1 CEP170 CEP131 MAPT CTNNA1 FLNC TNKS1BP1 LEO1 DYNC1LI1 CRYAB MTUS1 ANK2 CBX3 CD2AP ADD1 HECW2 MAP1A MYOZ2 SPTAN1 SNTA1 FRMD5 RABL6 MYH7 SVIL UBR4 CCAR2 TLN2 NUP93 SYNPO ABLIM3 AKAP12 LMNA EML3 PRKAR2A BAG3 TACC2 SIPA1L3 CDC42EP2 CEP76 PLEC MYH6 AKAP9 BIRC6 FHOD3 PEAK1 NEBL MYH4 PDLIM4 WAPL ANK3 RAB11FIP5 EHBP1L1 AHNAK LDB3 |
| **Molecular functions** | | | | | |
| Enrichment FDR | nPhosphoproteins | Pathway Proteins | Fold Enrichment | Pathway | Proteins |
| 0.002074 | 10 | 165 | 5.206505207 | Actin filament binding | CTNNA1 ITPRID2 MYH7 SVIL TLN2 ABLIM3 MYH6 FHOD3 NEBL MYH4 |
| 2.03E-05 | 18 | 342 | 4.521438732 | Actin binding | LIMCH1 CTNNA1 ITPRID2 FLNC MYOZ2 SPTAN1 MYH7 SVIL TLN2 SYNPO ABLIM3 PLEC MYH6 COBLL1 FHOD3 NEBL MYH4 LDB3 |
| 0.006425 | 10 | 198 | 4.338754339 | Transcription coactivator activity | NACA MED1 YAP1 TCF20 WWTR1 PHF2 CCAR2 TRIM28 SIN3A NUCKS1 |
| 0.006997 | 14 | 375 | 3.207207207 | GTPase activator activity | GIT2 ARHGAP31 SMCR8 DENND1B MON1A ARHGEF1 SIPA1L3 CDC42EP2 EEF1B2 GBF1 JUN RANBP3 DNMBP OBSCN |
| 3.6E-07 | 37 | 1014 | 3.134685827 | Protein-containing complex binding | ABCF1 GIT2 CEP131 RTN4 MAPT MED1 CTNNA1 ITPRID2 PHIP TNKS1BP1 CRYAB ANK2 CD2AP LMBRD1 HSPD1 IGF2R FRMD5 MYH7 SVIL TLN2 ABLIM3 BAG3 IRS2 MYH6 PEX1 FHOD3 ZC3H18 SIN3A H4F3 NEBL MYH4 LOC100911295 H1F2 NIPBL ZFP36 NAA10 KMT2D |
| 0.001012 | 20 | 560 | 3.06811914 | Protein domain specific binding | MYPN HDAC2 ARHGAP31 SQSTM1 ARHGAP5 MAPT MED1 TNKS1BP1 TJP1 CBX3 CD2AP TJP2 SNTA1 HSP90AB1 PRKAR2A TACC2 OSBP IRS2 TRIM28 NIPBL |
| 3.72E-05 | 28 | 809 | 2.973307052 | Cytoskeletal protein binding | MYPN LIMCH1 TOR1AIP1 MAPT CTNNA1 ITPRID2 FLNC CRYAB ANK2 MAP1A MYOZ2 SPTAN1 MYH7 SVIL TLN2 AGTPBP1 SYNPO ABLIM3 HSP90AB1 PLEC MYH6 COBLL1 FHOD3 NEBL MYH4 PDLIM4 OBSCN LDB3 |
| 0.00529 | 18 | 543 | 2.847756991 | Protein kinase binding | PRKAB1 SQSTM1 MAPT SMCR8 ANK2 TJP2 FRMD5 HSP90AB1 PRKAR2A LRBA IRS2 CCNYL1 MYH6 CAVIN2 PPP1R12C ZFP36 LDB3 KMT2D |
| 0.005955 | 19 | 605 | 2.697916334 | Kinase binding | PRKAB1 SQSTM1 MAPT SMCR8 ANK2 TJP2 FRMD5 HSP90AB1 PRKAR2A LRBA IRS2 CCNYL1 MYH6 CAVIN2 PPP1R12C PFKM ZFP36 LDB3 KMT2D |
| 1.33E-06 | 48 | 1677 | 2.45888618 | Enzyme binding | HDAC2 PRKAB1 ATP2A2 DNM1L SQSTM1 STX8 TOR1AIP1 RTN4 MAPT SMCR8 PDPK1 CHMP3 SLC9A1 TNKS1BP1 RAF1 DENND1B ANK2 CBX3 HSPD1 IGF2R TJP2 SNTA1 FRMD5 MCM2 CCAR2 AKAP12 HSP90AB1 PRKAR2A CDC42EP2 LRBA IRS2 CCNYL1 MYH6 CAVIN2 JUN PPP6R2 TRIM28 HACD3 FOXO4 PDLIM4 ALDOA PPP1R12C NIPBL PFKM ZFP36 LDB3 PPP1R3A KMT2D |

**Supplementary table 3:** This table provides a comprehensive overview of differentially phosphorylated proteins observed between NIPC and IPC groups. The data is specifically enriched in the top 10 pathways as determined by ShinyGO. Key metrics in the table include the false discovery rate (Enrichment FDR) indicating statistical significance, the count of detected proteins in the study (nProteins), the standard number of proteins typically observed in the pathway (Pathway Proteins), the degree of enrichment (Fold Enrichment), the categorization of the pathway, and a list of proteins associated with each pathway.
